# Status and Prospects of Botanical Biopesticides in Europe and Mediterranean Countries

**DOI:** 10.3390/biom12020311

**Published:** 2022-02-15

**Authors:** Fatma Acheuk, Shereen Basiouni, Awad A. Shehata, Katie Dick, Haifa Hajri, Salma Lasram, Mete Yilmaz, Mevlüt Emekci, George Tsiamis, Marina Spona-Friedl, Helen May-Simera, Wolfgang Eisenreich, Spyridon Ntougias

**Affiliations:** 1 Laboratory for Valorization and Conservation of Biological Resources, Faculty of Sciences, University M’Hamed Bougara of Boumerdes, Boumerdes 35000, Algeria; f.acheuk@univ-boumerdes.dz; 2 Clinical Pathology Department, Faculty of Veterinary Medicine, Benha University, Benha 13518, Egypt; shereenbh@yahoo.com; 3 Research and Development Section, PerNaturam GmbH, 56290 Gödenroth, Germany; Awad.Shehata@pernaturam.de; 4 Hochschule Trier, Schneidershof, 54293 Trier, Germany; katie1006dick@googlemail.com; 5 Laboratory of Molecular Physiology of Plants, Borj-Cedria Biotechnology Center, BP. 901, Hammam-Lif 2050, Tunisia; hajri_haifa@yahoo.fr (H.H.); salma.lasram.cbbc@gmail.com (S.L.); 6 Department of Bioengineering, Bursa Technical University, Bursa 16310, Turkey; mete.yilmaz@btu.edu.tr; 7 Department of Plant Protection, Faculty of Agriculture, Ankara University, Keçiören, Ankara 06135, Turkey; Mevlut.Emekci@agri.ankara.edu.tr; 8 Laboratory of Systems Microbiology and Applied Genomics, Department of Environmental Engineering, University of Patras, 2 Seferi St, 30100 Agrinio, Greece; gtsiamis@upatras.gr; 9 Bavarian NMR Center, Structural Membrane Biochemistry, Department of Chemistry, Technical University of Munich, Lichtenbergstr. 4, 85747 Garching, Germany; marina.spona-friedl@tum.de; 10 Institute of Molecular Physiology, Johannes Gutenberg-University of Mainz, 55128 Mainz, Germany; may-simera@uni-mainz.de; 11 Department of Environmental Engineering, Democritus University of Thrace, Vas. Sofias 12, 67132 Xanthi, Greece

**Keywords:** botanical insecticides, botanical herbicides, botanical fungicides, Mediterranean region, bioactive substances, mechanism of action

## Abstract

Concerning human and environmental health, safe alternatives to synthetic pesticides are urgently needed. Many of the currently used synthetic pesticides are not authorized for application in organic agriculture. In addition, the developed resistances of various pests against classical pesticides necessitate the urgent demand for efficient and safe products with novel modes of action. Botanical pesticides are assumed to be effective against various crop pests, and they are easily biodegradable and available in high quantities and at a reasonable cost. Many of them may act by diverse yet unexplored mechanisms of action. It is therefore surprising that only few plant species have been developed for commercial usage as biopesticides. This article reviews the status of botanical pesticides, especially in Europe and Mediterranean countries, deepening their active principles and mechanisms of action. Moreover, some constraints and challenges in the development of novel biopesticides are highlighted.

## 1. Introduction

**Figure 1:**
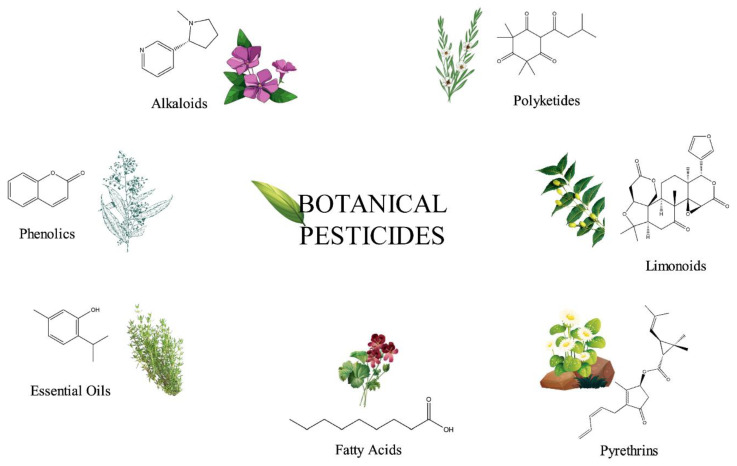
Structural diversity of bioactive compounds in plant-based pesticides.

To maximize food production for feeding the ever-increasing human population, a remarkable growth in the agrochemical market has been recorded worldwide. This increased demand has resulted in the development and wide acceptance of synthetic agrochemicals for managing crop pests and weeds. Plant protection products, such as synthetic insecticides and herbicides, have helped to maintain and increase agricultural yields for a long time. However, the use of chemical pesticides has also had numerous negative effects on human health and the environment [1,2,3,4]. The emergence of resistant insects and weeds still underscores the urgent need for novel and safe products. In developing countries, pesticides and herbicides are still frequently used in agriculture without control. These examples highlight the increasing demand for organic products and alternative eco-friendly approaches to substitute some of the synthetic pesticides [5]. For many years, botanical pesticides have been considered as gained alternatives to synthetic pesticides, due to their limited risk for the environment and humans. It has not been determined exactly when humans began to use plants and their metabolites as pesticides against insects and microbes, but it is already related to the onset of agriculture [6,7]. In Europe and North America, botanical pesticides have been applied for more than 150 years, much earlier than the discovery of the major classes of synthetic pesticides [8]. In Africa, the use of several plants, due to their suppressive activity to pests, has a centuries-long tradition passed down through the generations [9,10]. In modern agriculture, some botanical pesticides have already been registered in managing different crop pests, such as neem oil and pyrethrins. The benefits from the use of biopesticides include their low persistence and residuality, preventing environmental pollution and minimizing adverse effects on living organisms [11]. They can exhibit high host specificity, resulting in a delayed knockdown, and they are less prone to pest resistance [11]. Their highly versatile chemical structures (for examples, see Figure 1) arise from the enormous biosynthetic capabilities of plants.

In the first part of the present review, the structural basis and mechanistic principles of the currently used botanical insecticides, herbicides and fungicides in Europe and Mediterranean countries are summarized. In the second part, recent challenges and prospects in developing novel biopesticides are discussed.

## 2. Bioactive Principles in Botanical Pesticides

Botanical pesticides (“botanicals”) are characterized by bioactive mixtures/extracts/compounds from plant materials, which serve as insecticides and repellents but also as bactericides, fungicides, herbicides and nematicides [8]. In retrospect, the selection of effective botanical pesticides was carried out either through (i) testing the efficiency of traditional pesticidal plant extracts or substrates and identifying their active compounds or (ii) by a targeted or untargeted screening of some plant families selected after survey, which were then subjected to chemical analysis of their potential active compound(s) [12].

The industrial development and production of new botanical pesticides are challenging and, e.g., require (i) availability of pesticidal plant resources in a sustainable and large-scale manner, (ii) standardization in the processing of phytochemical substrates and performance of quality assurance protocols, and (iii) authorization approval, which implies extended record data on the environmental fate of the bioactive substrates and possible toxic effects on non-target organisms. 

Moreover, the combined use of biopesticides and biofertilizers can improve soil health status and prevent environmental pollution, thereby promoting sustainable agriculture [13]. Yadav et al. [14] reported that the adoption of organic farming practices through combined application of biofertilizers and biopesticides increases sustainability in plant cultivation. The use of biopesticides in combination with natural enemies can also enhance crop production [15]. The detailed mechanisms of these combinations are less known, but compatibility interactions among various biocontrol agents that act synergistically appear to be key parameters to improve plant protection in the future [16].

**Figure 2:**
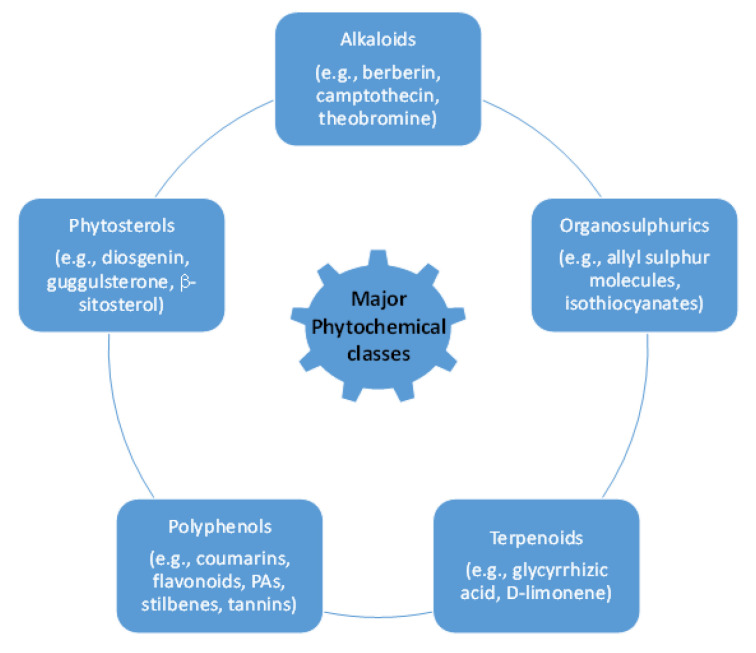
General classification of phytochemicals.

The active principles of botanical pesticides, especially the unique structural motifs of secondary metabolites, e.g., alkaloids, essential oils including terpenes, flavonoids, phenolics, phytosterols and polyketides as well as resins, are qualified to confer antibacterial, antifungal, herbicidal and insecticidal action [17,18] (Figure 1). Essential oils (EOs) and plant extracts are the botanical products most frequently used as biopesticides. Their action relies on plant-synthesized molecules as part of their intrinsic defensive mechanism against microbial pathogens and pests [19]: (i) To afford EOs, steam distillation is the most frequently applied method [20], but they can also be obtained from plants by fermentation, solvent extraction and enfleurage [21]. The obtained EO is a hydrophobic concentrate comprising of volatile chemical compounds, including terpenes, and others, namely alcohols, aldehydes, esters, fatty acids, ketones, phenols, as well as nitrogenous and sulphuric compounds [22]. (ii) Plant extracts are typically obtained from dried plant material, essentially by a solid/liquid extraction method using aqueous or organic solvents, e.g., acetone, ethanol, hexane or methanol [23]. These extracts are relatively complex mixtures of biomolecules in a liquid or semi-solid state or, after having removed the solvent, in a dry state. The typical bioactive compounds in biopesticidal extracts belong to secondary metabolites of plants, such as alkaloids, saponins or sterols [18,24] (Figure 2).

### 2.1. Alkaloids

**Figure 3:**
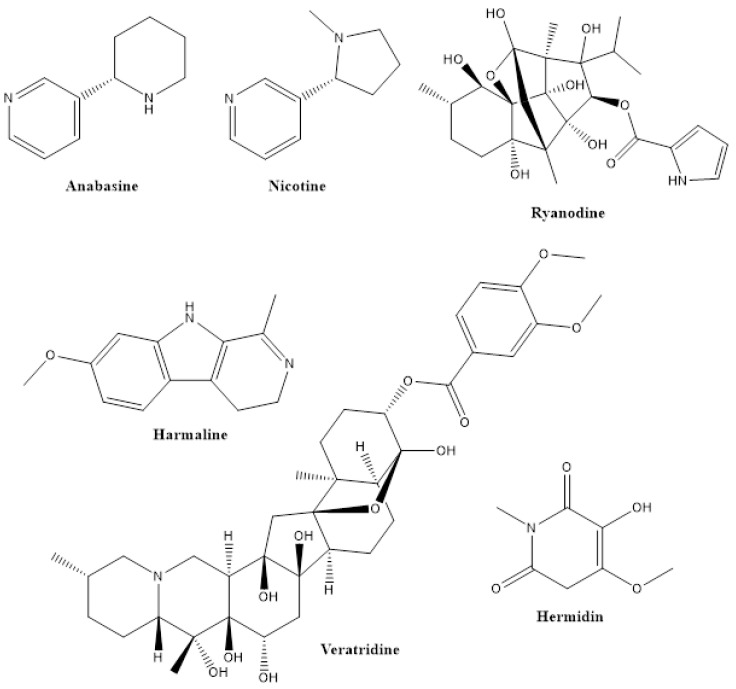
Structures of some pesticidal alkaloids.

Alkaloids, with more than 12,000 structures, form an exceptionally broad group with highly diverse chemical structures. Therefore, a structural definition of alkaloids is difficult, but criteria are (i) the low or medium molecular mass and the presence of one or more heterocyclic nitrogen-containing rings derived from amino acids in their molecule and (ii) the ability to provide an alkaline reaction in aqueous solution [25]. Alkaloids are found in considerable quantities in several plant species belonging to Annonaceae, Apocynaceae, Fabaceae, Fumariaceae, Lauraceae, Papaveraceae, Rubiaceae, Rutaceae and Solanaceae [26] and are accumulated in the aerial part of these plants [27]. Among many other properties, most alkaloids exhibit insecticidal activities at low concentrations. Typical examples of insecticidal alkaloids are anabasine (from *Anabasis aphylla*), nicotine (from *Nicotiana* species), ryanodine (Rayania; from *Ryania speciosa*) and veratridine (from *Schoenocaulon officinale*) [28] (Figure 3).

### 2.2. Phenolics

**Figure 4:**
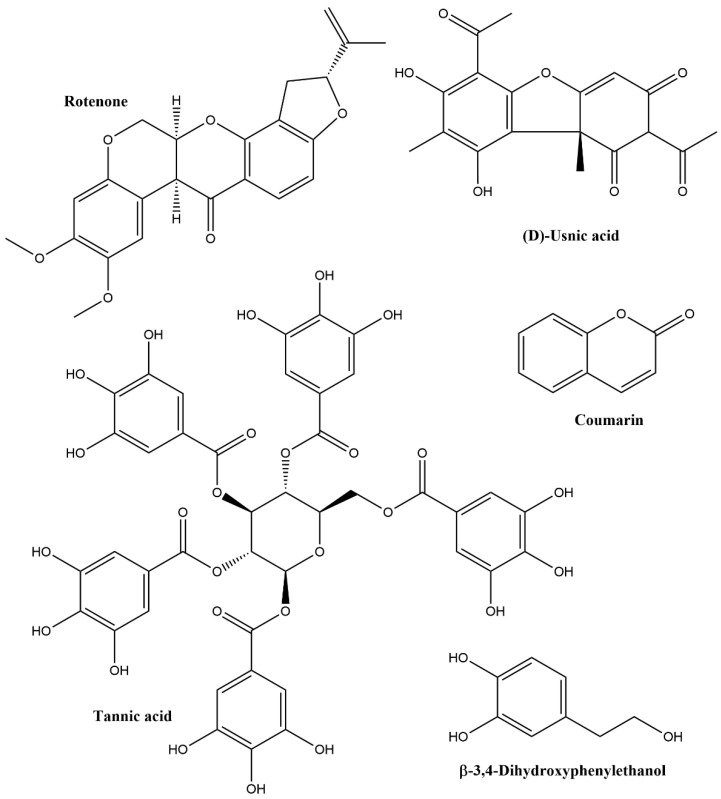
Structures of some pesticidal phenolics and O-heterocyclic compounds.

Phenolics are a highly heterogeneous group of plant secondary metabolites, identifying more than 50,000 distinct structures. Typical examples for pesticidal phenolics are shown in Figure 4. Their structures can be either simple (phenol: MW 94, identified in some plant EOs) or complex, e.g., polyphenols, including anthocyanins (MW up to 2000) and tannins (MW up to 20,000), identified in plant extracts. They can be further divided into flavonoids (anthocyanidins, flavones, flavonols, flavanones, isoflavones, coumarins and rotenoids) and non-flavonoids (phenolic alcohols, phenolic acids, stilbenes and lignans) [26,28,29,30]. Phenolics are involved in the attraction of pollinators as well as in the protection of plants from ultraviolet (UV) radiation, microbial invasion, and herbivore species [31,32]. According to Furiga et al. [33], phenolic compounds with antifungal properties include anthraquinones with different modes of action, coumarin and its derivatives, flavanols, flavonoids, simple-structured phenols, and tannins.

### 2.3. Essential Oils

**Figure 5:**
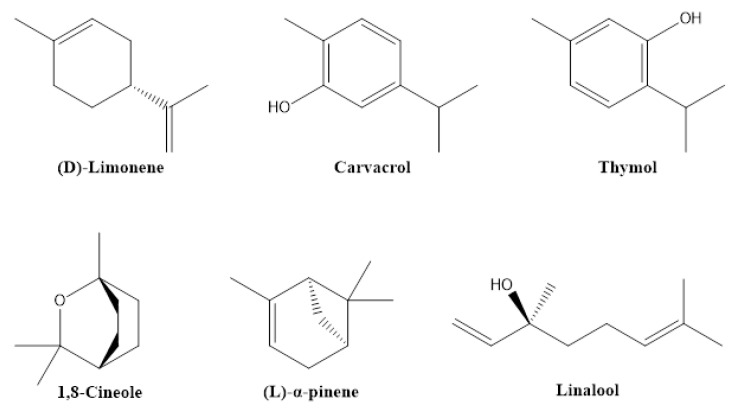
Structures of some pesticidal terpenes.

Essential oils (EOs) are derived from more than 17,500 known aromatic plants, mainly belonging to angiosperms, e.g., Asteraceae, Lamiaceae, Myrtaceae, Rutaceae and Zingiberaceae [34]. They can be obtained from the flowers, leaves, roots or seeds of these plants mainly by hydrodistillation. These distillates have a long history of usage in the perfume and food industry, mainly due to the sensory properties of the obtained volatile compounds. However, many compounds in these mixtures also exhibit pesticidal activities, mainly due to their insecticidal and repellent properties. These active principles include terpenes, such as 1,8-cineole (eucalyptol), β-caryophyllene, linalool, D-limonene, α-pinene, α-terpineol, thymol, carvacrol, and α-thujone (Figure 5), which can be obtained, for example, from orange oil, *Lavandula angustifolia*, *Origanum majorana*, *Rosmarinus officinalis*, *Salvia officinalis*, *Cannabis sativa*, *Tanacetum vulgare*, and *Thymus vulgaris* [8,35,36]. EOs are attractive due to easiness in preparation and chromatographic analysis, and due to the broad number of plant species synthesizing this cocktail of volatile phytochemicals of potential pesticidal usage [37].

### 2.4. Limonoids

**Figure 6:**
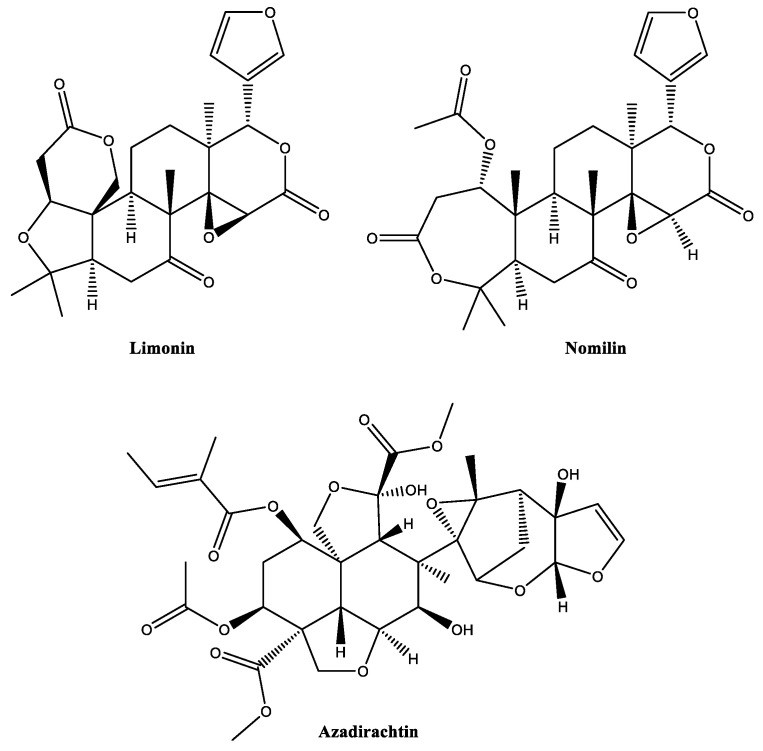
Structures of insecticidal limonoids.

The neem tree *Azadirachta indica* (Meliaceae) produces a great variety of phytochemicals, including alkaloids, fatty acids, polyphenols, saponins, terpenes and terpenoids [38,39]. Its most famous constituent, azadirachtin (Figure 6), is an active principle in many commercial bioinsecticides (Table S1). The UV- and acid-labile compound is a tetranortriterpenoid, which belongs to the class of limonoids. Generally, limonoids show complex structures, most of them carrying four six-membered carbon rings and a furanolactone-type moiety (for example, limonin and nomilin) (Figure 6). Limonoids have been identified, especially in the plant order of Sapindales (e.g., Meliaceae and Rutaceae families) and the family of Cucurbitaceae. For example, azadirachtin can be isolated from all parts of *A. indica*, especially from the seeds, with reported concentrations of 4–6 mg/g seed [40].

### 2.5. Pyrethrins

**Figure 7:**
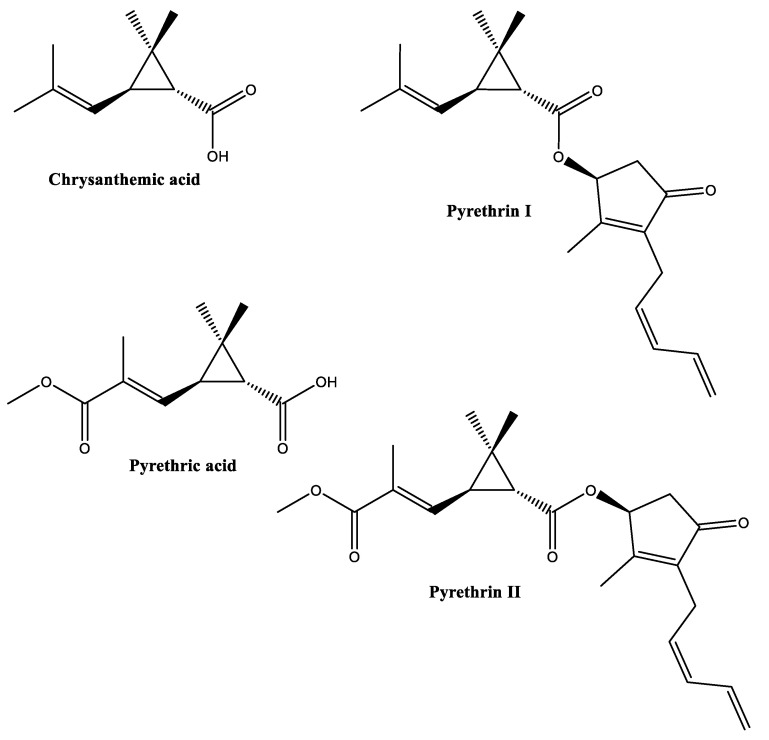
Structures of some pyrethrins.

Dalmatian pyrethrum (*Tanacetum cinerariifolium*), also called pyrethrum daisy, produces a potent insecticide, commonly named pyrethrin. Commercially, pyrethrin represents the most exploited bioinsecticide with a long history of successful applications against various insect pests [41] (see Table S1). Pyrethrin is a natural mixture comprising at least six compounds categorized into two groups, namely “Pyrethrins I” (pyrethrin I, cinerin I, jasmolin I; esters of chrysanthemic acid) and “Pyrethrins II” (pyrethrin II, cinerin II, jasmolin II; esters of pyrethric acid) [42,43] (Figure 7). Pyrethrins I and Pyrethrins II are plentiful in *Tanacetum*, especially in the flower heads (10–30 mg/g dry weight) [44].

### 2.6. Polyketides

**Figure 8:**
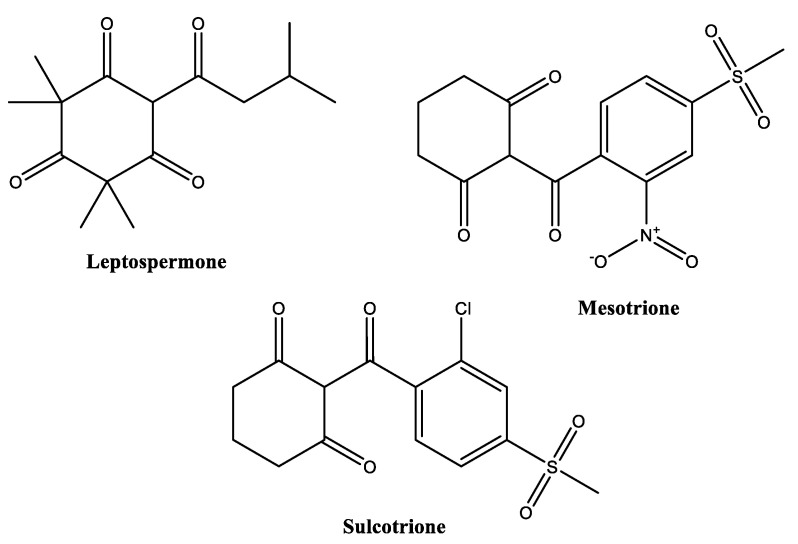
Structures of some herbicidal β-triketones.

Polyketides, e.g., β-triketones (Figure 8), represent another abundant family of biopesticides. They are biosynthesized from acetyl-CoA units by the action of polyketide synthases. A typical representative of a plant polyketide is the β-triketone leptospermone, which is produced by Myrtaceae spp., such as *Leptospermum scoparium* (manuka). β-Triketones can act as herbicides by inhibition of 4-hydroxyphenylpyruvate dioxygenase (HPPD), an enzyme that is relevant in the plant metabolism of tyrosine and the production of its downstream products.

### 2.7. Fatty Acids

**Figure 9:**
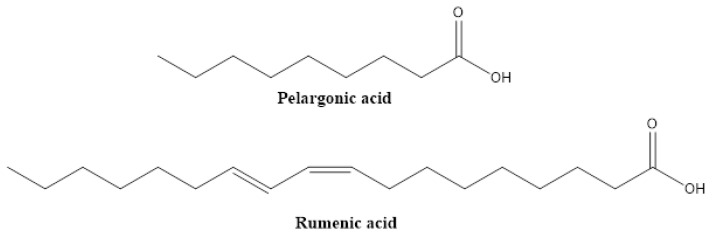
Structures of some pesticidal fatty acids.

Lipids and fatty acids typically serve as solvents that, in conjunction with emulgators, stabilize the active principles (such as azadirachtin or pyrethrins) in commercial biopesticides. However recently, conjugated unsaturated fatty acids, such as rumenic acid, which can be described as a conjugated linoleic acid (CLA) (Figure 9), have been shown to directly act on insect pests, such as the Colorado potato beetle. Foliar application of a mixture of CLAs demonstrated its insecticidal properties, inducing larval mortality, antifeedant effects and reduced survival rates of the eggs [45]. As another example, pelargonic acid from, e.g., *Pelargonium roseum* showed post-emergent herbicidal effects towards different broadleaf and grassy weeds, for example, in *Abutilon theophrast*, *Avena fatua*, *Brassica napus*, *Chenopodium* spp. and *Portulaca oleracea* [46,47,48,49].

## 3. Botanical Insecticides

### 3.1. State of the Art

It is well established that botanical insecticides can repel the attacking insects, inhibit their food ingestion, reduce their growth at various developmental stages, inhibit egg-laying activities, or even directly kill the insects by irreversible inhibition of one or more essential reactions in their metabolism. There is a wealth of literature documenting the insecticidal properties of plants extracts and isolates therefrom [12,34,50,51,52]. For example, Babaousmail and Isman [53] listed over 60 studies carried out in 1995–2015 that assessed the insecticidal properties of botanicals in North Africa, targeting insects from the orders of Coleoptera, Diptera, Hemiptera, Lepidoptera and Thysanoptera. Despite these abundant promising results, botanical insecticides produced in North African countries have not yet been exploited for use in the European market [6,17,50,53,54,55]. Nevertheless, recent changes in European Union regulations have renewed the interest in these findings [54,56].

Along with these studies, especially relevant for Mediterranean countries, our knowledge about the molecular mechanisms and bioactivities of plant derivatives, such as neem (from *Azadirachta indica*), pyrethrins (from *Chrysanthemum* or *Tanacetum*) and various plant EOs to arthropod pests, has expanded greatly over the last 20 years [6,57]. The modern technologies in analytics and biological sciences can now also be used to identify and optimize novel insecticides rationally.

Driven by the rapidly increasing knowledge about the modes of action of botanical insecticides, a considerable number of commercial products has been developed and registered for the European market (Table S1) [58]. Examples of commercially available botanical insecticides of wide use are neem oil from *Azadirachta indica* and pyrethrins from *Tanacetum cinerariifolium* [51]. However, the active principles present in most of these products rely on only a few compounds, namely azadirachtin, pyrethrins, fatty acids, and EOs. Notably, still little knowledge exists on scale-up production and applications of these few principles [6].

The same holds true for biopesticides in Africa, with a few products on the market (Table S2), but in these countries, on-farm applications of pesticidal plants by resource-limited smallholder farmers is a common practice [54]. The use of botanical insecticides by low income farmers is mostly based on the preparation and application of home-made aqueous extracts [59]. For example, aqueous extracts from neem leaves or seeds were successfully tested against Hemipterans, Lepidopterans and Thysanopterans, resulting in superior performance as compared to controls, probably reflecting the effects of azadirachtin and other limonoids present in *Azadirachta indica* [59]. In field experiments, Patil and Nandihalli [60] controlled mites by applying neem aqueous extract and oil. Moreover, Degri and Sharah [61] evaluated the performance of neem oil emulsions against fruit flies in the field. Complete protection against bean weevil during cowpea storage was achieved by the application of tobacco aqueous extracts [10]. Other home-made bioinsecticides commonly used are garlic extracts due to the presence of allicin, extracts of chili peppers due to high capsaicin content, mother of cocoa, due to the synthesis of coumarins, and clove basil, due to its essential oils [59]. 

Finally, only less than 1% of all plant secondary metabolites have been examined against insects. Moreover, only a few or even one insect species have been typically used for evaluation of the bioactive compounds [62]. This underpins the great potential to discover new bioinsecticides from plants in the future. 

### 3.2. Modes of Action of Botanical Insecticides

**Figure 10:**
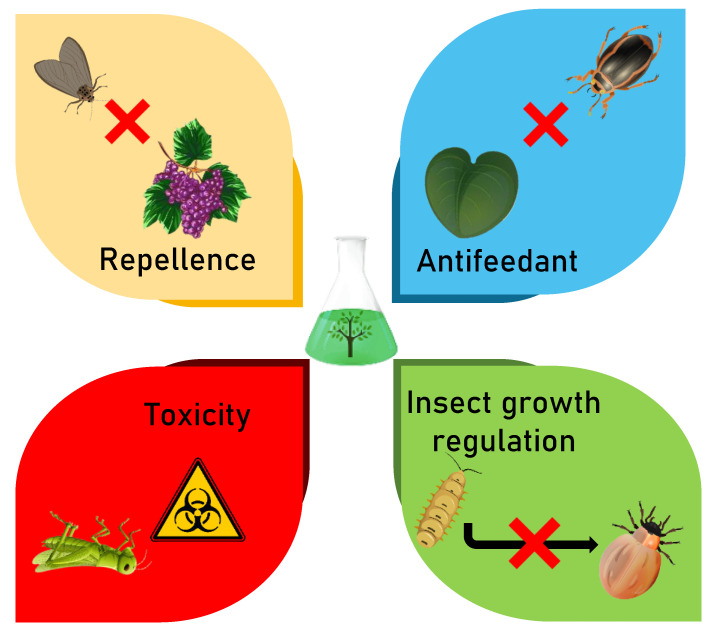
Mode of action of plant-based insecticides.

Botanical insecticides can induce various modes of action on the target pest species, including repellence, growth inhibition and modifications in their structure and physiology (Figure 10). On this basis, botanical insecticides represent promising alternatives in present and future pest management. The modes of action of some already established bioinsecticides are summarized in Table 1.

Botanical insecticides affect insect behaviour, physiology, morphology and metabolism [8,28,63], including growth and oviposition inhibition, ovicidal activities and the release of growth-reducing triggers [64,65,66,67,68,69]. Specifically, many of them exhibit neurotoxic mechanisms via interference with the neuromodulator octopamine or with GABA-gated chloride channels [8,70]. Among the different developmental stages of insect pests, adults are typically more sensitive to botanical insecticides, followed by larvae, pupae, and eggs [58].

***Alkaloids:*** Many alkaloids interfere with nerve acetylcholine receptors (e.g., nicotine) or membrane sodium channels (e.g., veratridine) of the insects. It is well known that alkaloids also can exert a feeding deterrent action against numerous insects, such as *Choristoneura fumiferana* [71] and *Spodoptera littoralis* [72]. Other alkaloids such as harmaline and hermidine again affect the growth and development of insects, including *Tribolium castaneum*, but their modes of action are not fully understood [73]. Ryanodine, an alkaloid compound from the plant species *Ryania speciosa*, exerts a strong insecticidal activity, acting at the sarcoplasmic reticulum, with ryanodine receptors influencing the secretion of Ca^2+^ [74]. Moreover, sabadilla alkaloids are agonists of Na^+^ channels in a similar manner to pyrethrins, causing neurotoxic effects on insect pests [75].

***Essential oils:*** Their mode of action virtually depends on the main constituents present in the respective plant oil as well as the targeted insect pest. Generally, it ranges from repellent and antifeedant effects to neurotoxic effects [8,63] but also includes other effects, such as growth and oviposition inhibition, ovicidal activities and growth-reducing triggers on a variety of insects [64,65,66,67,68,76]. The acute activity against various pests is characteristic of a predominant neurotoxic mode of action, while interference of oils with the neuromodulator octopamine or with the GABA-gated chloride channels is evidenced [8,70]. However, the detailed biochemical mechanisms and synergisms triggered by essential oils are incompletely understood.

***Phenolics and O-heterocyclic compounds:*** Plant phenolics and related compounds such as coumarines or anthraquinones are considered as an important defensive line against insects [28,77,78,79]. They reduce insect activities through deterrent or antifeeding effects. A large range of insects belonging to various orders, including aphids, Coleoptera, Diptera, Lepidoptera and Orthoptera, appear to be sensitive [80]. At the protein level, they provide inhibitory action on hydrolytic enzymes, such as pectinases, cellulases and proteases. They can also inhibit the production of hydrolytic enzymes and the biosynthesis of parasitic toxins. Conversely, they cause membrane alterations and inhibitions of the electron transport chain [81].

***Limonoids:*** The impacts of limonoids (e.g., azadirachtin) include antifeedant and/or physiological effects [38]. Azadirachtin is a growth regulator that disturbs the hormone system of insect pests by contact or ingestion [82]. The main mode of action is interference with the endocrine system of insects, which leads to a disruption of the synthesis of ecdysteroids (moulting hormones) and juvenile hormones. Moreover, limonoids block the release of morphogenetic peptide hormones, such as the prothoracicotropic hormone (PTTH) from the corpora cardiaca [38,40,63]. As a result, abnormal or delayed moults that are incompatible with the insects’ lifestyle and reduced growth can be seen upon azadirachtin treatment in many different insect species. Moreover, growth regulatory (IGR) effects due to azadirchatin can result in growth reduction, mortality increase, sterility, abnormal/delayed moults, and interference with cellular and metabolic processes (such as protein and hormone synthesis) [40,83,84]. In contrast to other botanical insecticides as well as to synthetic chemical insecticides, azadirachtin seems to act as a plant stimulant, which can lead to higher (crop) yields [85]. 

***Pyrethrins:*** Pyrethrins exhibit a neurotoxic mode of action by interfering with the ion channels of the insects, which are kept open and thus cause nerve impulses to fail [8]. In particular, pyrethrins interfere with the Na^+^, K^+^ exchange pump in nerve cells of target pests, leading to rapid nerve impulses and causing paralysis [86]. This results in a rapid knock-down effect in the insects and leads to death. If applied at low doses, pyrethrins also act as repellents on flying insects [42,87]. Many studies have proven the great efficacy of pyrethrins against various insect pests belonging to different orders, while their—albeit moderate—toxicity against mammals and non-target insect species provides some constraints for their broad usages [41].

#### 3.2.1. Repellent Effects

Repellents are substances that act from a distance and repel the insects from the treated plants or stored crops [8,88]. Due to their properties, natural repellents have been used for centuries in many countries. In recent years, several studies have convincingly demonstrated the repellent activities of plant products, including EOs (for review, see [89]). For example, EOs extracted from Asteraceae species, such as *Achillea millefolium*, *Artemisia absinthium*, *Santolina chamaecyparissus*, *Tanacetum patula* and *T. vulgare*, exhibited anti-settling activity when used against *Myzus persicae* females (green peach aphids) [90], and EOs from aniseed, lemongrass and peppermint were repellent against *Rhopalosiphum padi* [91].

Nevertheless, there are drawbacks in relation to the use of EO repellents, especially due to their volatility, low water solubility and oxidizability. However, the use of these compounds in nanoparticles could solve, to some extent, such limitations by reducing their degradation rate and increasing their residuality through evaporation prevention [92]. 

#### 3.2.2. Antifeedant Effects

Antifeedants are defined as compounds that “reduce consumption by an insect” or as “a peripherally mediated behaviour modifying substance (i.e., acting directly on the chemosensilla in general and deterrent receptors in particular) resulting in feeding deterrence” [93]. There is a strong thought that various plants remaining unattacked by insects possess a high content of antifeedant compounds [94]. In the 1970s and 1980s, the idea of applying insect antifeedants (“feeding deterrents”) gained ground by demonstrating the feeding deterrent effects of azadirachtin and neem seed extracts to numerous pest species [8]. However, antifeedant effects are not restricted to azadirachtin but include many terpenes and terpenoids [95], as well as phenolics and flavonoids [96]. As an example, Zhang et al. [97] reported that ginsenosides, triterpenoid saponines from *Panax ginseng*, possess potent antifeedant activities against *Pieris rapae*. Singh and Kaur [98] revealed that saponins act as insecticidal compounds due to their high toxicity to insect pests. Akhtar et al. [99] reported that naphthoquinones are effective feeding deterrents to the cabbage looper *Trichoplusia ni*.

Different molecular mechanisms may be involved in these antifeedant effects. It has been assumed that phenolic compounds inhibit important enzymes, such as proteases and other digestive hydrolases, and polyphenol oxidases (PPOs) [100], thereby decreasing the digestibility of nutritional proteins [101]. For example, multiple antifeedant actions of *Calceolaria integrifolia* were attributed to inhibition of phenol oxidase, proteinase or tyrosinase, to cuticle synthesis inhibition and to moulting sclerotization toxicity [102]. Some mechanisms by which compounds from bitter gourd extracts repel insects and alter their physiology were explained in a recent study [103]. It was found that transgenic flies with impaired aversive taste sensitive neurons exhibited a decreased aversion when exposed to bitter gourd extract, indicating that the bitter-sensitive gustatory neurons depend on these compounds.

#### 3.2.3. Toxic Effects 

The toxic effects of plant bioactive compounds are quite complex and rely on the chemical composition, the kind of insect pest, and the developmental stage of the insect [58]. In particular, the toxicity of plant EOs or extracts is mainly associated with the receptors and channels in the nervous system of insects, e.g., by affecting their γ-aminobutyric acid (GABA)-gated chloride and sodium channels, acetylcholinesterase (AChE), nicotinic receptors for acetylcholine (nAChR), octopamine and tyramine receptors [34,58,63].

AChE is a crucial enzyme in terminating the nerve impulse through the hydrolysis of neurotransmitters. Approximately 70% of the world market for insecticides is based on synthetic AChE inhibitors (organophosphates, carbamates and, more recently, neonicotinoids), including those insecticides acting on the voltage-gated sodium channels (in particular, pyrethrins) [104]. In recent years, a new area of biopesticides development was the detection of less harmful (for humans, other mammals, and the environment) natural compounds acting on insect nAChR by inhibiting their AChE activity.

Several EOs from aromatic plants, mostly monoterpenes, were shown to act as efficient AChE inhibitors in various insects [28]. More specifically, 1,8-cineole, carvone, linalool, α-pinene and phenolic compounds exhibited great toxicity via AChE inhibition [105]. Fenchone, S-carvone and linalool followed by estragole were also shown to efficiently inhibit AChE of stored-product pests under in vitro conditions [106].

The toxicity of alkaloids is also based on their anti-cholinesterase activity in the central nervous system, on disrupting cell membranes by interacting with the 3β-hydroxysterols of the membranes, and on modulating the active transport of ions through membranes, finally leading to metabolic dysfunction [107]. As an example, the alkaloids berberine, palmatine and sanguinarine were shown to substantially affect AChE, choline acetyl-transferase, butyrylcholinesterase, alpha 1- and alpha 2-adrenergic, nicotinergic, muscarinergic and serotonin-2 receptors in *Periplaneta americana* via interaction with nAChRs [28].

In this context, synergistic effects (“entourage effects”) could occur when dealing with complex mixtures of bioactive constituents, for example, EOs or plant extracts containing alkaloids plus terpenes or fatty acids. These mixtures are qualified to multiply the desired insecticidal activities. This effect should also not be underestimated in preventing resistance against pathogens and pests. Taking advantage from the entourage effect is probably one of the most important benefits of future biopesticides based on botanical compound mixtures [63,108,109].

#### 3.2.4. Growth Regulation Effects: Larval Growth and Adult Reproduction

Plant secondary metabolites can confer properties similar to synthetic growth regulators, such as teflubenzuron [110,111]. Acting as insect growth regulators (IGRs), phytochemicals influence the reproduction, development and metamorphosis of insects. These effects can cause irreversible changes in their physiology and behaviour [112,113]. Numerous bioactive compounds in plant extracts can affect the endocrine regulation of moulting and metamorphosis and thereby act as IGRs. It was postulated that they probably have juvenile hormone analogous (JHAs)-like properties [104].

As examples, extracts from *Calceolaria talcana* and *Condalia microphylla* were found as efficient IGRs, with similar activity to phytoecdysteroids, as indicated by their strong inhibition of the moulting process [114,115]. Their action is similar to juvenile hormone mimics [115]. The effect of azadirachtin on the growth and developmental stages of larvae with interference on ecdysone and juvenile hormone regulation has also been documented in various insects [116]. Muema et al. [117] suggested that morphological aberrations in mosquito larvae associated with IGRs from *Zanthoxylum chalybeum* are due to impacts on the ecdysteroid pathway, finally inducing larval retardation.

Quiroz-Carreño et al. [118] reported that the benzylisoquinoline alkaloids coclaurine, laurolitsine, boldine and pukateine could interact with the heterodimer ecdysone receptor. Endocrine disturbance could also be found in non-emergent adults or deformed adults (adultoids), probably triggered by plant extracts containing phytoecdysteroids or terpenoids that act as JHAs [119].

Many previous studies reported that some EOs and plant extracts from various species have strong effects on the reproduction of insects by reducing adults’ weight, the longevity of females, fecundity and fertility, and egg hatchability, or by increasing neonate larval mortality [120,121]. IGRs can also change metabolic pathways, leading to ultrastructural and morphological malformations, modifying the duration of larval, pupal, and imaginal development, showing deterrent activity and finally leading to insect death [122]. For example, a decrease in oviposition of *Damalinia limbata* was detected in neem-exposed female lice. Abdellaoui et al. [123] reported that the application of olive leaf extract (OLE) resulted in decreased fecundity and fertility and limited oocyte growth during the first gonadotropic cycle. Furthermore, OLE reduced the protein, lipid and carbohydrate content of ovaries, indicating a disruption in the embodiment of the haemolymph substrates in the oocytes and interference with the vitellogenic process.

These examples underline that reproduction and hormonal regulation in insects are key targets for the development of novel insecticides [124]. Acquired resistance to plant-derived IGRs is extremely difficult to be developed by insects since these compounds quite efficiently mimic the natural insect hormones [125]. Thus, plant compounds acting as IGRs may provide the most promising source for the development of novel bioinsecticides [126].

#### 3.2.5. Metabolic Effects

Many of the effects described above are also associated with a reprogramming of the core carbon and nitrogen metabolism of the insects. For example, toxic effects due to inhibition of hydrolytic enzymes or effects upon hormonal signalling cascades finally end in changes of substrate usages and/or the rerouting of metabolic fluxes. It appears that metabolic targets are under-represented in the classes of insecticides and, therefore, have high potential for the discovery of novel bioinsecticides based on new modes of action.

In support of this hypothesis, a recent study using metabolomics investigated the effect(s) of azadirachtin on *Helicoverpa armigera* larvae [83]. It was revealed that the levels of most metabolites were remarkedly affected, underlining the complete reprogramming of the core metabolism. Similarly, protein levels were changed. Those proteins associated with immunity, RNA processing, and protein synthesis were upregulated, while proteins associated with amino acid storage, defence mechanisms, energy transfer and lipid metabolism were downregulated [83]. This seminal study also showed that *H. armigera* is unable to metabolize azadirachtin; thus, the insect could not neutralize the toxic effects of azadirachtin. Another study reported a significant reduction of the quantity and relative composition of fatty acids as well as the downregulation of carbohydrate metabolism in *Bactrocera dorsalis*, due to azadirachtin treatment [84].

## 4. Botanical Herbicides

### 4.1. State of the Art

Several extracts from plants have been described to exhibit herbicidal activities (Table S3). The genus *Syzygium*, family Myrtaceae, contains various species, such as *Syzygium aromaticum*, syn. *Eugenia caryophyllus* [127,128,129]. The main phytotoxic compounds from *Syzygium* sp. (clove essential oil) are β-caryophyllene, eugenol, and eugenol acetate [128,129,130,131]. For example, eugenol inhibited the root growth of *Avena fatua* [132], *Abutilon theophrasti*, *Amaranthus* spp., broccoli (*Brassica oleracea*), *Chenopodium album* [133,134], *Portulaca oleracea*, and *Urtica urens* [134]. Additionally, it inhibited the seedling growth of *Chenopodium album*, *Melilotus indicus*, *Raphanus raphanistrum*, *Sisymbrium irio* [131], *Amaranthus retroflexus*, and *Brassica oleracea* [133]. Both clove oil and particularly eugenol exhibited herbicidal effects on broccoli leaf, although the presence of leaf epicuticular wax greatly reduced the retention of these oils [133]. Several bioherbicides based on clove oil are now available (Table S4).

The genus *Cymbopogon*, family Poaceae, contains more than 140 species of tropical and subtropical plants cultivated in Asia, South America, Australia and Africa [135]. Several species, such as *Cymbopogon citratus*, *C. nardus* and *C. winterianus*, exhibit phytotoxic effects. The phytotoxic effect of *Cymbopogon*-derived herbicides is attributed to the presence of citronellal, geraniol and citronellol and are well documented against germination of wheat seeds, *Ageratum conyzoides*, *Amaranthus tricolor*, *Cassia occidentalis*, *Cenchrus echinatus*, *Chenopodium album*, *Digitaria horizontalis*, *Malvastrum coromandelianum*, *Parthenium hysterophorus*, and little seed canary grass [136], as well as *Amaranthus thaliana* and *Senecio jacobaea* leaves [137].

The genus *Cinnamomum*, family Lauraceae, is an important fragrant spice plant containing more than 250 evergreen trees [138,139]. The commonly known cinnamon being of Indian, Australian and Asian origin is cultivated currently in West Indies, South America and further tropical climates [138]. Species of cinnamon include *Cinnamomum verum*, *C. zeylanicum* and *Cinnamon cassiacae* [138,140]. The main bioactive substances of cinnamon essential oil are eugenol and trans-cinnamic aldehyde [137,138,141]. Cinnamon exhibited herbicidal effects on *Ageratum conyziodes*, *Amarantus retroflexus*, *Ambrosia artemisiifolia*, *Bidens pilosa*, *Cassia occidentalis*, *Chenopodium album*, *Commelina benghalensis*, *Echinochloa crus-galli*, *Leptochloa chinensis*, *Lolium* spp., *Phalaris minor*, *Sinapis arvensis*, *Sorghum halepense*, and *Taraxacum officinale* [132,142,143,144]. Cinnamon essential oil also inhibited the seed germination of *Amaranthus tricolor* and *Echinochloa crus-galli* [137,145].

The genus *Eucalyptus*, family Myrtaceae, is a plant originating from Australia, which is currently cultivated in subtropical areas and in the Mediterranean region and includes more than 800 species [146]. Various species, such as *Eucalyptus camaldulensis*, *E. citriodora* and *E. globulus*, were reported to exhibit herbicidal effects [146,147,148]. The harmful effects of eucalyptus essential oil were affirmed for several plants, for example, *Cassia occidentalis*, *Lolium rigidum*, *Portulaca oleracea*, *Vicia sativa* [149,150], *Celtis occidentalis* and *Echinochloa crus-galli* [147], while causing inhibition of seed germination of *Lactuca sativa*, *Pisum sativum*, *Triticum durum*, *Zea mays* [151], *Acroptilon repens* and *Portulaca oleracea* [152].

*Leptospermum* is a member of the Myrtaceae family that originated from Australia and New Zealand [153,154]. It contains about 87 species, including *L. lanigerum*, *L. liversidgei*, *L. nitens*, *L. polygalifolium*, *L. scoparium*, *L. speciosum* and *L. whitei* [153,154,155,156,157]. The herbicidal activity of *Leptospermum scoparium* (manuka) is caused by high concentrations of herbicidal β-triketones, especially leptospermone (see Figure 8) [156,158,159,160]. Manuka oil inhibited the seedling growth of *Amaranthus powellii*, *Digitaria sanguinalis*, *Eleusine coracana*, pepper, sweet corn and tomato [159,161]. Since there is only a small post-emergent effect of Manuka oil on some crops, low doses can be used as selective herbicides, especially in pepper, sweet corn and tomato field [159,161]. To the best of our knowledge, no bioherbicides containing Manuka oil as an active ingredient are currently offered.

The genus *Ocimum*, family Lamiaceae, is an herbaceous plant that thrives in India, Thailand and the Mediterranean area [162]. This family contains about 160 species, including *Ocimum basilicum*, *O. gratissimum* and *O. sanctum* [163,164]. The main components that are responsible for the phytotoxic impacts on plants are, for example, geranial and geraniol, linalool, methyl cinnamate and methyl eugenol [164,165,166,167,168]. The herbicidal effect of *Ocimum basilicum* and *O. tenuiflorum* was documented for *Echinochloa crus-galli*, *Lactuca sativa*, *Lepidium sativum*, *Lolium multiflorum*, *Medicago sativa*, and *Phleum pretense*, as well as for seeds of *Amaranthus* spp., *Cucumis sativus*, *Glycine max* (soybean), *Portulaca oleraceae*, *Zea mays* (maize), and *Solanum lycopersicum* [144,166,169,170]. *Ocimum* spp. are more effective on post-emerged seeds and young weeds than on pre-emerged seeds [166], suggesting that application in the field before germination of non-target plants could be propitious. To confirm this strategy, a better understanding of the phytotoxic mechanisms is needed. To the best of our knowledge, no commercial bioherbicides based on basil essential oil are available.

The genus *Origanum*, family Lamiaceae, originated in the Mediterranean basin, particularly from Spain and France. *Origanum onites*, *O. vulgare* subsp. *hirtum*, *O. vulgare* subsp. *vulgare* and *O. vulgare* subsp. *virens* are the most common species of this family [171,172]. *Origanum vulgare* essential oil has a high content of carvacrol, γ-terpinene and thymol [171,173,174]. Carvacrol, as the main compound in *Origanum vulgare* essential oil, has antigerminative functions against *Alcea pallida*, *Amaranthus retroflexus*, *Capsicum annuum*, *Centaurea solstitalis*, *Chenopodium album*, *Echinochloa crus-galli*, *Lactuca sativa*, *Lolium perenne*, *Portulaca oleracea*, *Raphanus raphanistrum*, *Rumex crispus*, *R. nepalensis*, and *Sinapis arvensis* [144,149,173,175,176,177,178]. The application of *Origanum onites* and *O. vulgare* caused inhibition of germination of *Avena sterilis*, *Cucumis sativus*, *Sinapis arvensis*, *Solanum lycopersicum*, and a number of wheat cultivars [149,169,171,179,180]. Although there are many publications on the inhibitory effect of *Origanum vulgare*, as of now, no commercial bioherbicides based on Oregano essential oil or carvacrol are available.

The genus *Pelargonium*, family Geraniaceae, has origins from India, Pakistan, and South Africa [181,182,183]. It contains 250 species, including *Pelargonium graveolens*, *P. reniforme*, and *P. sidoides* [182]. *P. graveolens* has a high content of essential oil [184,185], with citronellol, citronellyl formate and trans-geraniol being its main components [138,182,186,187]. The plant also contains pelargonic acid, showing post-emergent herbicidal effects towards different broadleaf and grassy weeds, for example, in *Abutilon theophrast*, *Avena fatua*, *Brassica napus*, *Chenopodium* spp., *Portulaca oleracea*, and many more [46,47,48,49]. Being a non-selective bioherbicide makes it unsuitable for use in plantations, but it could be used as a potential bioherbicide for a wide range of applications [47]. According to the European Food Safety Authority (EFSA), pelargonic acid has no harmful effects on human or animal health and is of relatively low risk to the environment [188]. Taking into account that the herbicidal effect and environmental compatibility were officially confirmed, several commercial bioherbicides based on pelargonic acid are now on the market (Table S4). However, to the best of our knowledge, no commercial bioherbicide based on *Pelargonium* essential oil is available.

The genus *Thymus* belongs to the Lamiaceae family and originates from Central and South Europe [138]. Examples from the 21 species cultivated, e.g., in Bulgaria, Romania and Iran, are *Thymus callieri*, *T. serpyllum*, and *T. vulgaris* [149,189,190]. Thyme essential oil has a high content of carvacrol and thymol, as well as borneol [171,174,190], which seem to be responsible for its herbicidal activity. Carvacrol shows post-emergent effects. It inhibited *Amaranthus retroflexus*, *Avena fatua*, *Echinochloa crus-galli*, *Erigeron bonariensis*, and *Portulaca oleracea* [48,191]. *Thymus* species also show pre-emergent allelopathic performance. *Thymus fontanesii* inhibited seed germination of *Avena fatua*, *Cyperus rotundus*, *Sinapis arvensis*, *Sonchus oleraceus*, and *Xanthium strumarium* [171]. *Thymus proximus* suppressed the seed germination of *Amaranthus retroflexus* and *Poa anuua* [192]. *Thymus algeriensis* inhibited *Medicago sativa* and *Triticum astivum* seedling growth [193]. Many other *Thymus* species also showed phytotoxic effects to further herbs [192], indicating that thyme essential oil, in general, has high herbicidal potential and can be used in a wide range of weeds. Thymol and carvacrol were shown to be more phytotoxic than glyphosate, referring to the root growth of *Echinochloa crus-galli* [191]. It also acted on some glyphosate-resistant weeds [48], suggesting that thyme essential oil could be used especially on glyphosate-resistant weeds, e.g., *Portulaca oleracea* [48].

The genus *Lavandula*, part of the Lamiaceae family, originates from the Mediterranean region but is also currently cultivated in other countries [194,195]. There are several species of lavender, for example *Lavandula angustifolia*, *L. latifolia*, and *L. spica* [195,196], showing a variation in terpene composition. *L. angustifolia*, for example, being an important *Lavandula* species, has a high content of linalool and linalyl acetate [194,197]. Further compounds are 1,8-cineole and fenchone [175]. Linalool worked pre- and post-emergent on *Cassia occidentalis* [198]. Certain *Lavandula* species showed allelopathic action in several plants. *Lavandula* spp. exhibited phytotoxicity against seeds of *Amarantus retroflexus*, *Lolium* spp., and *Sinapis arvensis* [142]. *Lavandula stoechas* and *L. angustifolia* reduced seed germination of *A. retroflexus* and *P. oleracea* [175]. *Lavandula x intermedia Emeric ex Loisel*. cv. Super A influenced the germination and seedling emergence of *A. retroflexus*, *Rumex crispus*, and *Sinapis arvensis* [199]. Remarkably, *Cicer arietinum*, *Helianthus annuus* cv. Sirena, *Solanum lycopersicum*, and *Triticum aestivum* cv. Gün-91 were not sensitive to lavender essential oil [175,199], indicating that *Lavandula* species could work as a selective bioherbicide in tomato fields and other cash crops [175]. To review if more cash crops react on treatment with lavender essential oil, further investigations are needed. To the best of our knowledge, no commercial bioherbicides are available that contain lavender essential oil.

### 4.2. Modes of Action of Botanical Herbicides 

***Eugenol:*** The phenylpropanoid eugenol is known to elicit the generation of active oxygen species in plants, which leads to cell membrane damage and inhibition of photosynthesis. The enhanced activity of ROS-scavenging enzymes inhibits further metabolic pathways [131,132,133,137,143,144]. 

***Cinnamic aldehyde:*** In contrast to eugenol, cinnamic aldehyde does not peroxidate lipids of the cell membrane but interacts with the integrated receptors on the surface, leading to manipulation of ligand-based metabolic pathways [137,200].

***Monoterpenes:*** The monoterpenes citronellal, citronellol and gerniol regulate the phospholipid synthesis in the cytoplasmic membrane during oxidative stress, altering membrane permeability and causing electrolyte losses [201]. Citronellal has a phytotoxic effect on both seed germination and plants. It inhibits respiration, photosynthesis, and other metabolic pathways. Correlating to other isoprene derivatives, citronellol releases reactive oxygen species that induce oxidative stress on plants. Electrolyte leakage, caused by disrupted cell membranes and reduced photosynthesis, are examples of manipulated metabolic pathways that harm the target weed [137,183]. Citronellol inhibited seed germination of *Ageratum conyzoide*, *Amaranthus virdis* and *Cassia occidentalis* [198,202,203]. Citronellal and citronellol as pure substances proved to be more effective than the mixture in *Cymbopogon* essential oil [136,137]. Negative synergism between citronellal and citronellol and possibly other ingredients can be assumed. Notably, the phenylpropanoid cinnamic aldehyde seems to respond faster on target plants than for citronellal and citronellol [137], suggesting that cinnamon essential oil is a quickly working herbicide compared to citronella essential oil. Therefore, mixing of lemongrass oil with other essential oils for potential synergistic effects needs further investigations. The phytotoxicity of eucalyptus essential oil mainly refers to the monoterpene 1,8-cineol, causing oxidative stress followed by membrane disruption and electrolyte leakage [131,146,204]. It inhibits mitosis by inhibiting G1 phase and several enzymes as p38, resulting in further metabolic interceptions, for example, in photosynthesis and energy metabolism [148], which lead to cellular damage and death of the target plant [205]. It was found that 1,8-cineol alone has only poor phytotoxic effects and increases its adverse effects when combined with other components [150]. Therefore, it needs further investigations to find out which combinations of terpenes or essential oils obtain the largest herbicidal effects. 

The monoterpenes carvacrol, γ-terpinene, and thymol are known to disrupt the cell membrane and to influence several metabolic pathways, photosynthesis, cell respiration and mitosis [171,206]. However, there is not much known about the exact mechanism of carvacrol in plant metabolism [137], but it seems that this monoterpene deploys cytotoxic effects [191,207] that harm several plants. Similar to other monoterpenes, carvacrol has intense inhibitory effects on germination and seedling growth [179], suggesting that pathways other than photosynthesis are induced by carvacrol. Moreover, a release of reactive oxygen molecules and resulting reprogramming of metabolic pathways could be assumed. 

Linalool is a highly potent monoterpene, affecting several metabolic pathways. Similar to other monoterpenes, linalool reduces photosynthesis, respiratory activity and alteration of water status [198]. Further ROS-connected manipulations of the plant’s metabolism, such as enzyme inhibition and lipid oxidation, can be assumed [202].

***Pelargonic acid:*** Pelargonic acid has a strong allelopathy effect [137,208] with even higher herbicidal effects compared to citronellol [48,137].

***β-Triketones:*** Herbicidal β-triketones, such as leptospermone from *L. scoparium*, regulate carotenoid synthesis by inhibiting para-hydroxyphenylpyruvate dioxygenase (HPPD), an essential enzyme cofactor for plant defence systems [161]. Accordingly, Manuka oil causes oxidative stress [209], which subsequently reduces photosynthesis and finally leads to electrolyte leakage and to the death of the plant [161]. Leptospermone in Manuka essential oil has a long persistence in the soil and remarkable synergistic effects with other bioactive substances, leading to high efficiency in its herbicidal pre-emergent function [159,161]. Further studies are needed to explore the suitable concentrations and combination of Manuka and other essential oils. Although some essential oils, such as lemongrass and pine oil, display contact burn-down bioactivity, Manuka oil exhibited systemic activity as well as a synergistic effect with pelargonic acid [210]. Leptospermone has residual soil activity, suggesting its potential use as an organic herbicide [159].

## 5. Botanical Fungicides

### 5.1. State of the Art

Botanical fungicides have recently gained ground since they have less or no negative impact compared to synthetic antifungal agents, including residual effects and induced resistance. In this regard, they may be effective, selective, biodegradable or less toxic to the ecosystem [211]. However, still only a limited number of botanical fungicides has been authorized and commercialized [211] (Table S5).

Nevertheless, today’s farmers are increasingly aware of the hazards of synthetic fungicides and are looking for botanical alternatives. Thus, the demand for botanical fungicides is rapidly increasing, and there is ongoing research on the exploration of potential plants having fungicidal activities, the development of effective extraction methods, and the elucidation of the mode of action on target fungi. 

### 5.2. Modes of Action of Botanical Fungicides

***Phenolics:*** Research on botanical pesticides has shown that extracts from various plants exerted activity against various phytopathogenic fungi without imposing negative effects. According to Lattanzio et al. [212], the antifungal properties of phenolics are attributed to their lipophilicity and/or the occurrence of the hydroxyl groups in their structure. Due to their binding properties to adhesions and proteins, they are qualified to disrupt membranes, inactivate enzymes and complex metal ions, thereby exhibiting toxic effects upon fungi (Table 2) [213]. In particular, the lipophilicity of phenolics facilitates penetration of the cytoplasmic membrane, whereas hydroxyl groups are involved in the uncoupling of oxidative phosphorylation. 

***Terpenes:*** To date, several terpenes have proven to be active against a wide variety of fungal species [214]. Mendoza et al. [215] revealed that terpenes cause membrane disruption in fungi, owing to their lipophilic properties. However, these compounds can also induce structural alterations on hypha and mycelia, thereby lowering the production of toxins, e.g., aflatoxin and fumonisin produced by *Aspergillus* and *Fusarium* species, respectively, resulting in reduced pathogenicity of mycotoxin-producing fungi [216].

***Alkaloids:*** Zhou et al. [217] have shown by in vitro assays using plant extracts from *Veratrum taliense* that the verazine and jerveratrum-type alkaloids exhibit strong suppressive properties against the phytopathogenic oomycete *Phytophthora capisis*. This antioomycete activity is due to the fact that these alkaloids intercalate into the cell wall and/or DNA [218].

## 6. Regulation of Biopesticides in the European Union

Directive 67/548/EEC on products that might affect human health and Directive 78/631/EEC on plant protection products were the main legislative documents providing rules on the use of pesticides. In the EU, the authorization of plant protection agents initially relied on Directives 91/414/EEC and 98/8/EC, concerning the placing of plant protection and biocidal products on the market, respectively, as amended in Regulation (EU) No. 528/2012 on the authorization of biocides. Directive 91/414/EEC was further amended by Directives 2001/36/EC and 2005/25/EC to include regulatory issues regarding microbial biological control agents. Moreover, minimum requirements for biological control agents used in organic farming are stated in Regulation (EU) No. 889/2008. Both agrochemicals and biopesticides are enclosed under the term “plant protection products”.

Meanwhile, the Common Agricultural Policy (CAP), which regulates the use of pesticides and give guidelines for more sustainable practices in the agricultural sector, the Water Framework Directive (WFD), Regulation (EU) No. 396/2005 on upper residue limits of pesticidal products in food and feed, the Waste Framework Directive, the Directive on hazardous waste and the Directives on health and the safety of workers and on the preservation of the biodiversity also affected the use of pesticides. Moreover, the Directives for wild birds and habitats and on the preservation of the biodiversity proliferate the use of biopesticides. Directive 1999/45/EC, on the classification and labelling identity of harmful chemical preparations, should also be taken into consideration. In addition, specific strategies were taken place in the Sixth Environmental Action Programme of the European Council and Parliament to promote the sustainable use of pesticides through a thorough analysis of the subject, focusing on identifying pressures on the environment and on the selection of appropriate technological and law-based tools to face ecological concerns, including legislative proposals, communication actions and impact assessment reports (https://ec.europa.eu/environment/archives/ppps/pdf/pesticides_en.pdf accessed on 14 December 2021).

Directive 2009/127/EC, amending Directive 2006/42/EC, “with regard to machinery for pesticide application”, Directive 2009/128/EC on the Sustainable Use of Pesticides, Directive 2005/25 providing the evaluation principles for microbial plant protection products, as incorporated in Regulation (EU) No. 546/2011, Regulation (EU) No. 2017/1432, amending Regulation (EU) No. 1107/2009 concerning market placement of plant protection agents, Regulation (EU) No. 396/2005 “on maximum residue levels of pesticides” and Regulation (EU) No. 284/2013 on the data requirements for phytoprotective products are the main drivers in the authorization and use of plant protection products [219,220]. However, despite the important progress in the field of biological control agents, no revision of data requirement for low-risk bioactive products was performed in Regulation (EU) No. 284/2013 from those reported in Directive 2001/36/EC [221]. The plant health Directive 2000/29/EC also influences the regulation status of biopesticides, whereas Regulation (EU) No. 1143/2014 on Invasive Alien Species affects the authorization for non-indigenous organisms [222]. Regulation (EU) 1107/2009, which is the backbone in authorization process of biopesticides, permits the option of registering biorationals either as “low-risk” or “basic” substances, following the procedure described in article 22 and 23 of this Commission document, respectively, providing two registration routes to SMEs (small- and medium-sized enterprises) [223].

The EU Reflection Paper “Towards a Sustainable Europe by 2030” and the United Nations 2030 Agenda and its Sustainable Development goals set as a priority the sustainable use of pesticides (file:///C:/Users/Asus/Downloads/NA0219035ENN.en.pdf accessed on 14 December 2021). A response of the Commission Green Deal commitment towards sustainable practices is the “Farm to Fork” and the “Biodiversity” strategies, promoting a reduced use of agrochemicals, a fact that can promote biopesticide market growth. According to the evaluation report of Regulations (EU) No. 1107/2009 and No. 396/2005, “The aim of EU legislation on pesticides is therefore not to eliminate pesticides but rather to minimize their impact on human health and the environment through reduced dependency on pesticides, alternative methods and through increased use of low risk and non-chemical pesticides” [224], thus supporting initiatives for reducing dependency on chemical substances. In previous evaluations of the Regulation on plant protection products, the need for further protecting the environment and human and animal health, together with the necessity for enhanced transparency and independence of science, was denoted. The second evaluation report also stated the need for more incentives and research on low-risk solutions, whereas it required the reinforcement of the precautionary principle and the implementation of hazard-based methods for authorization. Moreover, prevention and monitoring of harmful organisms through protection actions, preference on non-chemical approaches for pest management and reduction of pesticides levels and facing resistance issues are the main topics of Directive 2009/128/EC [225].

## 7. Current Challenges in the Development and Registration of Biopesticides

There is less doubt that biopesticides have great potential to control insects and vector-borne diseases, bacterial and fungal pathogens, and weeds. An expansion of biopesticides over conventional pesticides is observed globally, with the global biopesticides market size exceeding USD 4 billion at the outset of this decade, expecting to double by the year 2025, with bioinsecticides being approximately half of the total biopesticide share [226]. More than 1400 biopesticide registrations have been made worldwide [227], although a much lower number of registrations are considered in Europe due to the complex regulatory system in the EU. Besides, more than 200 biopesticide products are currently available in the North American market, compared to approximately 60 in the EU [228].

Pyrethrum and azadirachtin are the main biopesticidal compounds in the global biopesticide market, with pyrethrum representing ca. 80% of botanical insecticides worldwide. Kenya is the leading country in pyrethrum production, providing ca. 70% of the global share [229]. Regarding azadirachtin from neem seeds, India is a major neem oil producer with more than 2.5 lakh tones [229].

Regulation stringency in using chemical pesticides, environmental and safety considerations, and new technological achievements are among the factors boosting biopesticide consumption and global market share. Besides, many countries in North Europe and North America have possessed the political willingness in recent years to reduce conventional pesticides by strengthening biopesticide consumption through restrictions in the use of synthetic pesticides and investments in biological agents [230]. Conversely, the wide use of biopesticides is still restricted compared to synthetic pesticides, due to lower acute activity and higher degradation rate, higher production complexity, poor investment performance, restricted formulation approaches, and past reputations for poor efficiency [231]. Moreover, regulations are still a drawback for the promotion of biopesticides in many countries. Thus, a rapid and straightforward registration process and effective communication within registrants and authorities through easiness in exchanging knowledge and information and improving organization can facilitate the expansion of the global biopesticides market. Apart from the high demand for organic products in the USA, the simplification of the registration submission process and the reduction in registration fees by the US Environmental Protection Agency (US EPA) resulted in increased numbers of registrations of biopesticides (https://www.epa.gov/sites/default/files/2015-08/documents/biopesticide-oversight-chapter_0.pdf accessed on 14 December 2021), being a good paradigm to follow by the other countries [231]. The fact that the registration of such products in countries such as the United States of America is less expensive and needs less time to prepare the application file and to obtain the registration certificate compared to synthetic pesticides have also contributed to the penetration of the biopesticides to pesticides markets outside the EU. 

A concern has arisen regarding the low number of SMEs registering plant protection products in Europe due to high requirements for authorization, with the stakeholders to emphasize the disproportionation in authorization occurring for low-risk substrates, such as biopesticides. Several researchers argue that European legislation on pesticides was initially designed for chemical substrates and not for the approval of biopesticides since the latter have distinct properties compared to synthetic pesticides [232]. Moreover, segmentation and differences in the authorization process of biological agents are obstacles to the registration in a higher number. Regarding biofertilizers, including microbial biostimulants, their regulation relies on Regulation (EU) No. 2019/1009 on fertilizing products, while the authorization of biocides is based on Regulation (EU) No. 528/2012 [223]. Moreover, Regulation (EU) No. 2018/848 “on organic production and labelling of organic products”, replacing Council Regulation (EU) No. 834/2007, handles issues regarding the authorization of organic farming [233]. 

Enforcement of policies promoting natural biological products through legislative acts can play a pivotal role in boosting the biopesticides market. For instance, a ban on the use of chemical pesticides within the limits of cities in some municipalities led the federal government of Canada to include such measures in the Pest Control Products Act, as the consequence of the public’s awareness to abate pesticide application [230], creating new opportunities for biopesticides market to cover the gap.

The adoption of biopesticides as reduced-risk products and the harmonization with registration practices promoting biopesticide use as those followed by US EPA and proposed by OECD can increase the relatively low number of commercially available biological products, which is among the main drawbacks for the expansion of this safer pest management approach [234]. Immature attempts in the past to develop novel biological agents, mainly restricted to laboratory attempts of Universities’ consortia, and the still low share of biopesticides in the pesticide market (less than 3%) [235] prevent large pesticide companies from expanding their investment in this field. Besides, incompatibility in their laboratory infrastructure and lack of expertise in microbial formulation and fermenter technology affects their shift into developing biopesticide products. Easiness in the authorization process can facilitate the further penetration of SMEs into the biopesticides market, resulting in an increased number of bioactive products, simultaneously reducing the cost of production, which is among the major obstacles in farmers’ acceptability [236]. Regarding farmers, the effectiveness and reliability of biopesticides as compared to conventional agrochemicals are among the major criteria for consideration. Conversely, SMEs that are interested in biopesticides commercialization often present limitations in the infrastructure, investment capital and scale-up knowledge. Financial support, facilitation in preparing registration dossier and an up-to-date decision system is worth providing at early development stages by the governmental and registration authorities. Moreover, an integrated plan to inform growers of biopesticides availability, uses and advantages should be issued by public authorities and private companies. Thus, collaboration and interaction among farmers, SMEs, key pesticide companies, universities, consumers and authorities are needed to enhance building capacity in the field of biopesticides development and to ensure the scale-up and quality assurance of the novel biological products. OECD reports that an increasing number of global initiatives have taken place to provide the legislative framework towards a shift from chemical pesticide-based cultivations to more sustainable integrated pest management systems, which have lower health risks for farmers and consumers, and which have reduced environmental impact [237].

Conversely, the wide biodiversity of pesticidal plant species in Africa, which are commonly used by low-income farmers to manage pests, suggests the existence of a new market segment that caters to both resource-poor farmers and organic producers in developed countries [5]. As an example, world pyrethrum production mainly relies on plant species commercially cultivated in Africa and specifically in Kenya, which covers 80% of global production [238]. Stevenson, Isman and Belmain [54] reported that several indigenous pesticidal plant species are used in the Mediterranean region, including North Africa, where the application of botanical pesticides with activity against arthropod pests has been confirmed. In the last decade, an important number of scientific reports have focused on optimizing the application of pesticide plant species at a smallholder farmer level, especially in Africa. 

Isman and Grieneisen [56] reported that the major obstacles for a broad adoption of plant-derived pesticidal products are (i) the lack of scale-up paradigms in international literature, (ii) the limited number of low-cost effective plant pesticidal products, (iii) the strict legislative framework for authorization, (iv) the short persistence of many botanical pesticides during application due to either evaporation or microbial degradation, and (v) the variation in phytochemical composition of plant extracts and mixtures due also to diverse climatic conditions that influence the extraction process, a fact that can result in the production of pesticidal products of weaker activity. Sufficient quantities of pesticidal plants at consistent availability should be guaranteed for scale-up development of botanical insecticides. In addition, the recent achievements in chemical synthesis and extraction, biochemical engineering, biotechnology, and molecular biology (e.g., DNA recombinant technologies) can boost the development and commercialization of novel plant pesticidal products [58].

Based on the current status of EU legislation on biopesticides, the following conclusions can be drawn:

- Biopesticides and related products should be evaluated in a more biological and ecological context.
- A simplification of authorization should be enacted to enhance the further penetration of biopesticides into plant protection markets, shifting agriculture to more sustainable integrated pest management systems.
- There is a need for unification of legislation for low-risk biologically based plant protection products, separating their evaluation from conventional chemical pesticides, with a focus on food safety, human health and protection of the environment.
- Financial and in-depth scientific support through research programmes should be provided to facilitate SMEs to develop more biological control products and key pesticides producers to switch to more sustainable products.
- Support of networking approaches and effective links within farmers, SMEs and the industry will further stimulate the biopesticides market.

## Figures and Tables

**Table 1:** Modes of action of some bioinsecticides.

Insecticide	Main Mode of Action	Remarks
Azadirachtin	Stops larval moulting by inhibiting ecdysteroids synthesis (moulting hormones). Acts as repellent and antifeedant. Causes sterility in adult females.	Broad spectrum insecticide targeting aphids, whiteflies, fungus gnats and two-spotted mits.
Nicotinoids	Mimic acetylcholine neurotransmitter.	Banned by the EU in 2018, due to its harmful effect on honeybees.
Pyrethrins	Disruption of sodium and potassium ion exchange in insect nerve fibres, leading to immediate paralysis.	Synergized by piperonyl butoxide (PBO).
Ryanodine	Acts as stomach poison, with ryanodine receptors influencing the secretion of Ca^2+^.	Synergized by PBO.
Rayania	Inhibits cellular respiration (mitochondrial poison).	Extremely toxic to fish and insects. EU announced in 2008 a phase-out of rotenone (EC 2008/317).
Rotenone	Neurotoxic, causing paralysis and death.	Synergized by PBO or N-octyl bicycloheptene dicarboximide (MGK-264).
Sabadilla	Repellent, anti-feedant, Na^+^ channel agonist, neurotoxic.	Broad spectrum insecticide, mild activities, highly toxic to bees.

**Table 2:** Modes of action of phenolic and O-heterocyclic compounds on fungi.

Compound	Remarks
Flavonoids	Bind to adhesions
Phenol	Substrate deprivation
Phenolic acids	Membrane disruption
Quinones	Bind to adhesions link to cell wall, enzyme inactivation
Tannins	Bind to proteins, bind to adhesions, membrane disruption, enzyme inhibition, substrate deprivation and metal ions complexation

## Supplementary Materials

The following are available online at https://www.mdpi.com/article/10.3390/biom12020311/s1, Table S1: Botanical insecticides authorised in Germany, Table S2: Commercially available products based on botanical insecticides in Algeria (DPVCT 2015), Table S3: Plant-derived substrates and extracts with bioherbicide activity, Table S4: Selected commercial bioherbicides, Table S5: Commercially available plant-derived substrates and extracts with fungicide activity.
