# Supplementary material for: Status and Prospects of Botanical Biopesticides in Europe and Mediterranean Countries
Source: Biomolecules. 2022 Feb 15;12(2):311. doi: 10.3390/biom12020311 (PMC8869379; doi:10.3390/biom12020311)
Supplement: Supplementary file 1 [file biomolecules-12-00311-s001.zip › biomolecules-1545347-supplementary.pdf]

Supplementary Material

of

Status and Prospects of Botanical Biopesticides in Europe and Mediterranean Countries

Fatma Acheuk [1], Shereen Basiouni [2], Awad Shehata [3], Katie Dick [4], Haifa Hajri [5], Salma Lasram [5], Mete Yilmaz [6], Mevlüt Emekci [7], George Tsiamis [8], Marina Spona-Friedl [9], Helen May-Simera [10], Wolfgang Eisenreich [9,*] and Spyridon Ntougias [11,*]

[1] Laboratory for Valorization and Conservation of Biological Resources, Faculty of Sciences, University M'Hamed Bougara of Boumerdes, 35000 Boumerdes, Algeria; f.acheuk@univ-boumerdes.dz

[2] Clinical Pathology Department, Faculty of Veterinary Medicine, Benha University, Benha, Egypt; shereenbh@yahoo.com

[3] Research and Development Section, PerNaturam GmbH, 56290 Gödenroth, Germany; Awad.Shehata@pernaturam.de

[4] Hochschule Trier, Schneidershof, 54293 Trier, Germany; katie1006dick@googlemail.com

[5] Laboratory of Molecular Physiology of Plants, Borj-Cedria Biotechnology Center, BP. 901 Hammam-Lif, 2050 Tunisia; hajri_haifa@yahoo.fr; salma.lasram.cbbc@gmail.com

[6] Department of Bioengineering, Bursa Technical University, 16310 Bursa, Turkey; mete.yilmaz@btu.edu.tr

[7] Department of Plant Protection, Faculty of Agriculture, Ankara University, 06135 Keçiören, Ankara, Turkey; Mevlut.Emekci@agri.ankara.edu.tr

[8] Laboratory of Systems Microbiology and Applied Genomics, Department of Environmental Engineering, University of Patras, 2 Seferi St, 30100 Agrinio, Greece; gtsiamis@upatras.gr

[9] Department of Chemistry, Bavarian NMR Center, Structural Membrane Biochemistry, Technical University of Munich, Lichtenbergstr. 4, 85747 Garching, Germany; marina.spona-friedl@tum.de; wolfgang.eisenreich@mytum.de

[10] Institute of Molecular Physiology, Johannes Gutenberg-University of Mainz, 55128 Mainz, Germany; may-simera@uni-mainz.de

[11] Department of Environmental Engineering, Democritus University of Thrace, Vas. Sofias 12, 67132 Xanthi, Greece; sntougia@env.duth.gr

* Correspondence: WE: wolfgang.eisenreich@mytum.de; SN: sntougia@env.duth.gr

Table S1. Botanical insecticides authorised in Germany.

Product name	Manufacturer	Active Substance(s)	Insect Pest(s)
Austriebsspritzmittel	Solabiol	paraffin oil (CAS 8042-47-5)	scale insects, wolly aphids
Austriebs-Spritzmittel Para Sommer	Schopf Hygiene	paraffin oil (CAS 8042-47-5)	Aphids as virus vectors, scale insects
Bayer Garten Austriebsspritzmittel	Bayer CropScience	paraffin oil (CAS 8042-47-5)	scale insects, wolly aphids
Bayer Garten Bio Spinnmilben- & Schädlingsfrei AF	Bayer CropScience	pyrethrins + rapeseed oil	aphids, moth scale insects, sucking insects, scale insects, wolly aphids
Bayer Garten Bio-Schädlingsfrei AF	Bayer CropScience	pyrethrins + rapeseed oil	aphids, moth scale insects, sucking insects, scale insects, wolly aphids
Bayer Garten Bio-Schädlingsfrei Neem	Bayer CropScience	azadirachtin	potato beetle, leaf-mining insects, biting insects, sucking insects, aphids, free-feeding butterfly caterpillars, cockchafer, vine pest, white flies, fungus gnats, thrips
Bayer Garten Bio-Schädlingsfrei Akut AF	Bayer CropScience	pyrethrins + rapeseed oil	aphids, moth scale insects, sucking insects, scale insects, wolly aphids
Bayer Garten Orchideen- & Zierpflanzenspray Lizetan	Bayer CropScience	pyrethrins + rapeseed oil	aphids, moth scale insects, sucking insects, scale insects, wolly aphids
Bayer Garten Orchideen-Spray Lizetan AF	Bayer CropScience	pyrethrins + rapeseed oil	aphids, moth scale insects, sucking insects, scale insects, wolly aphids
Bayer Garten Schädlingsfrei Lizetan AZ	Bayer CropScience	azadirachtin	potato beetle, leaf-mining insects, biting insects, sucking insects, aphids, free-feeding butterfly caterpillars, cockchafer, vine pest, white flies, fungus gnats, thrips
Bayer Garten Zierpflanzen- & Rosen-Spray Lizetan AF	Bayer CropScience	pyrethrins + rapeseed oil	aphids, moth scale insects, sucking insects, scale insects, wolly aphids
Bayer GartenSchädlingsfrei Lizetan Gießmittel AZ	Bayer CropScience	azadirachtin	potato beetle, leaf-mining insects, biting insects, sucking insects, aphids, free-feeding butterfly caterpillars, cockchafer, vine pest, white flies, fungus gnats, thrips

Bio Spinnmilben- & Schädlingsfrei	Solabiol	pyrethrins + rapeseed oil	potato beetle, sucking insects, free-feeding butterfly caterpillars, biting insects, aphids, moth scale insects, scale insects, wolly aphids, leaf wasps, leaf-eating beetles
Bio Spinnmilben- & Schädlingsfrei AF	Solabiol	pyrethrins + rapeseed oil	aphids, moth scale insects, sucking insects, scale insects, wolly aphids
Buchsbaumzünslerfrei	Solabiol	azadirachtin	potato beetle, leaf-mining insects, biting insects, sucking insects, aphids, free-feeding butterfly caterpillars, cockchafer, vine pest, white flies, fungus gnats, thrips
BUCHSBAUMZÜNSLER-FREI ORGANIC	Schopf Hygiene	azadirachtin	potato beetle, leaf-mining insects, biting insects, sucking insects, aphids, free-feeding butterfly caterpillars, cockchafer, vine pest, white flies, fungus gnats, thrips
CELAFLOR Schädlingsfrei Neem	Evergreen Garden Care Deutschland GmbH	azadirachtin	potato beetle, leaf-mining insects, biting insects, sucking insects, aphids, free-feeding butterfly caterpillars, cockchafer, vine pest, white flies, fungus gnats, thrips
Celaflor Schädlingsfrei Obst & Gemüse	Evergreen Garden Care	rapeseed oil	aphids, white flies, scale insects
Celaflor Schädlingsfrei Rosen	Evergreen Garden Care	rapeseed oil	aphids, white flies, scale insects
Celaflor Schädlingsfrei Zierpflanzen Konzentrat	Evergreen Garden Care	rapeseed oil	aphids, white flies, scale insects, sucking insects
Chrysal Blattläuse Stop Pumpspray	Braun GmbH	potassium salts of natural fatty acids (= potash soap)	aphids, white flies
Chrysal Schildläuse Stop	Chrysal International BV	paraffin oil (CAS 8042-47-5)	scale insects, wolly aphids
COM 107 03 AI EC	COMPO GmbH	rapeseed oil	aphids, white flies, spider mites, scale insects, thrips, cicadas
COM 107 04 AI AL	COMPO GmbH	rapeseed oil	aphids, white flies, spider mites, scale insects, thrips, cicadas
COMPO Austrieb-Spritzmittel	COMPO GmbH	paraffin oil (CAS 8042-47-5)	scale insects, wolly aphids

COMPO BIO Insekten-frei Neem	COMPO GmbH	azadirachtin	potato beetle, leaf-mining insects, biting insects, sucking insects, aphids, free-feeding butterfly caterpillars, cockchafer, vine pest, white flies, fungus gnats, thrips
COMPO Blattlaus-frei Nativert	COMPO GmbH	rapeseed oil	aphids, white flies, scale insects, thrips, cicadas
COMPO Blattlaus-frei Nativert AF	COMPO GmbH	rapeseed oil	aphids, white flies, spider mites, scale insects, thrips, cicadas
COMPO Buchsbaumzünsler K.O.	COMPO GmbH	azadirachtin	potato beetle, leaf-mining insects, biting insects, sucking insects, aphids, free-feeding butterfly caterpillars, cockchafer, vine pest, white flies, fungus gnats, thrips
COMPO Grün- und Blühpflanzen Schädlings-frei AF	COMPO GmbH	rapeseed oil	aphids, white flies, spider mites, scale insects, thrips, cicadas
COMPO Insektenmittel PREV-AM	COMPO GmbH	Orange oil	white flies, sucking insects
COMPO Kräuter & Gemüse Blattlaus-frei Nativert AF	COMPO GmbH	rapeseed oil	aphids, white flies, spider mites, scale insects, thrips, cicadas
COMPO Nativert Blattlaus-frei	COMPO GmbH	rapeseed oil	aphids, white flies, spider mites, scale insects, thrips, cicadas
COMPO Nativert Blattlaus-frei AF	COMPO GmbH	rapeseed oil	aphids, white flies, spider mites, scale insects, thrips, cicadas
COMPO Nativert Kräuter & Gemüse Blattlaus-frei AF	COMPO GmbH	rapeseed oil	aphids, white flies, spider mites, scale insects, thrips, cicadas
COMPO Orchideen Schädlings-frei AF	COMPO GmbH	rapeseed oil	aphids, white flies, spider mites, scale insects, thrips, cicadas
COMPO Schädlings-frei plus	COMPO GmbH	pyrethrins + rapeseed oil	potato beetle, sucking insects, free-feeding butterfly caterpillars, biting insects, aphids, moth scale insects, scale insects, wolly aphids, leaf wasps, leaf-eating beetles
COMPO Schädlings-frei plus AF	COMPO GmbH	pyrethrins + rapeseed oil	aphids, moth scale insects, scale insects, wolly aphids, sucking insects
COMPO Trauermücken-frei	COMPO GmbH	azadirachtin	potato beetle, leaf-mining insects, biting insects, sucking insects, aphids, free-feeding butterfly caterpillars,

			cockchafer, vine pest, white flies, fungus gnats, thrips
Dr. Stähler Blattlausfrei-Spray	Schopf Hygiene	potassium salts of natural fatty acids (= potash soap)	aphids, white flies
Dr. Stähler Schädlingsfrei-Spray	Schopf Hygiene	pyrethrins + rapeseed oil	aphids, moth scale insects, scale insects, wolly aphids, sucking insects
Eradicoat	CERTIS EUROPE B.V.	maltodextrin	white flies, aphids
Eradicoat Max	CERTIS EUROPE B.V.	maltodextrin	white flies, spider mites
ETISSO Schädlings-frei EC	Frunol Delicia GmbH	azadirachtin	potato beetle, leaf-mining insects, biting insects, sucking insects, aphids, free-feeding butterfly caterpillars, cockchafer, vine pest, white flies, fungus gnats, thrips
FLiPPER	Bayer CropScience	fatty acids (C7-C20)	aphids, white flies
Herba-Vetyl flüssig	Vetyl-Chemie GmbH	pyrethrins + rapeseed oil	potato beetle, sucking insects, free-feeding butterfly caterpillars, biting insects, aphids, moth scale insects, scale insects, wolly aphids, leaf wasps, leaf-eating beetles
Kantaro	CERTIS EUROPE B.V.	maltodextrin	white flies, aphids
Lizetan AZ Schädlingsfrei	Solabiol	azadirachtin	potato beetle, leaf-mining insects, biting insects, sucking insects, aphids, free-feeding butterfly caterpillars, cockchafer, vine pest, white flies, fungus gnats, thrips
Lizetan AZ Schädlingsfrei Gießmittel	Solabiol	azadirachtin	potato beetle, leaf-mining insects, biting insects, sucking insects, aphids, free-feeding butterfly caterpillars, cockchafer, vine pest, white flies, fungus gnats, thrips
Lizetan Orchideen- & Zierpflanzenspray	Solabiol	pyrethrins + rapeseed oil	aphids, moth scale insects, scale insects, wolly aphids, sucking insects
Lizetan Orchideen-Spray AF	Solabiol	pyrethrins + rapeseed oil	aphids, moth scale insects, scale insects, wolly aphids, sucking insects

Lizetan Zierpflanzen- & Rosen Spray	Solabiol	pyrethrins + rapeseed oil	aphids, moth scale insects, scale insects, wolly aphids, sucking insects
Micula	Evergreen Garden Care	rapeseed oil	aphids, white flies, scale insects, thrips, cicadas
Naturen Austriebs-Spritzmittel	Evergreen Garden Care	rapeseed oil	aphids, white flies, scale insects, sucking insects
Naturen Bio Austriebs-Spritzmittel	Evergreen Garden Care	rapeseed oil	aphids, white flies, scale insects, sucking insects
Naturen Bio Schädlingsfrei Neem	Evergreen Garden Care	azadirachtin	potato beetle, leaf-mining insects, biting insects, sucking insects, aphids, free-feeding butterfly caterpillars, cockchafer, vine pest, white flies, fungus gnats, thrips
Naturen Bio Schädlingsfrei Obst & Gemüse	Evergreen Garden Care	rapeseed oil	aphids, white flies, scale insects
Naturen Bio Schädlingsfrei Zierpflanzen	Evergreen Garden Care	rapeseed oil	aphids, white flies, scale insects
Naturen Bio-Blattlausfrei	Evergreen Garden Care	rapeseed oil	aphids, white flies, scale insects
Naturen Bio-Schädlingsfrei	Evergreen Garden Care	rapeseed oil	aphids, white flies, scale insects, sucking insects
Naturen Bio-Schildlausfrei	Evergreen Garden Care	rapeseed oil	aphids, white flies, scale insects
Naturen Blattlausfrei	Evergreen Garden Care	rapeseed oil	aphids, white flies, scale insects
Naturen Schädlingsfrei	Evergreen Garden Care	rapeseed oil	aphids, white flies, scale insects, sucking insects
Naturen Schädlingsfrei Obst und Gemüse	Evergreen Garden Care	rapeseed oil	aphids, white flies, scale insects
Naturen Schädlingsfrei Obst und Gemüse Konzentrat	Evergreen Garden Care	rapeseed oil	aphids, white flies, scale insects, sucking insects
Naturen Schädlingsfrei Zierpflanzen	Evergreen Garden Care	rapeseed oil	aphids, white flies, scale insects
Naturen Schädlingsfrei Zierpflanzen Konzentrat	Evergreen Garden Care	rapeseed oil	aphids, white flies, scale insects, sucking insects
Naturen Schildlausfrei	Evergreen Garden Care	rapeseed oil	aphids, white flies, scale insects
Neem Bio-Schädlingsfrei	Solabiol	azadirachtin	potato beetle, leaf-mining insects, biting insects, sucking insects, aphids, free-feeding butterfly caterpillars, cockchafer, vine pest, white flies, fungus gnats, thrips

Neem Plus Schädlingsfrei	NEUDORFF GmbH KG	azadirachtin + rapeseed oil	aphids, scale insects, spider mites, thirps, white flies
NeemAzal-T/S	Biofa AG	azadirachtin	potato beetle, leaf-mining insects, biting insects, sucking insects, aphids, free-feeding butterfly caterpillars, cockchafer, vine pest, white flies, fungus gnats, thrips
Neudosan AF Neu Blattlausfrei	NEUDORFF GmbH KG	potassium salts of natural fatty acids (= potash soap)	aphids, white flies
Neudosan Neu	CERTIS EUROPE B.V.	potassium salts of natural fatty acids *(= potash soap)*	aphids, white flies, sucking insects
Neudosan Neu Blattlausfrei	NEUDORFF GmbH KG	potassium salts of natural fatty acids (= potash soap)	aphids, white flies, sucking insects
Neudosan Obst-& GemüseSchädlingsFrei	NEUDORFF GmbH KG	potassium salts of natural fatty acids (= potash soap)	aphids, white flies, sucking insects
Para Sommer	FMC Agricultural Solutions	paraffin oil (CAS 8042-47-5)	Aphids as virus vectors, scale insects
Pflanzen Paral Blattlaus-Frei S	Evergreen Garden Care	rapeseed oil	aphids, white flies, scale insects
Pflanzen Paral Schädlings-Frei S	Evergreen Garden Care	rapeseed oil	aphids, white flies, scale insects
Piretro Verde	Copyr S.p.A.	pyrethrins	crossed grape berry moth (hay and sour worm), single-barked grape berry moth (hay and sour moth), white flies, aphids, scale insects, leaf wasps, leaf-eating beetles, free-feeding butterfly caterpillars
PREV-AM	COMPO GmbH	Orange oil	white flies, sucking insects
PREV-GOLD	Oro Agri International	Orange oil	white flies, aphids
Promanal AF Neu Schild- und Wolllausfrei	NEUDORFF GmbH KG	paraffin oil (CAS 8042-47-5)	scale insects, wolly aphids
Promanal AF GrünpflanzenSchädlingsFrei	NEUDORFF GmbH KG	paraffin oil (CAS 8042-47-5)	scale insects, wolly aphids

Promanal Neu	NEUDORFF GmbH KG	paraffin oil (CAS 8042-47-5)	scale insects, wolly aphids
Promanal Neu Austriebsspritzmittel	NEUDORFF GmbH KG	paraffin oil (CAS 8042-47-5)	scale insects, wolly aphids
Promanal Neu Schild- und Wolllausfrei	NEUDORFF GmbH KG	paraffin oil (CAS 8042-47-5)	scale insects, wolly aphids
Pyreth Natur-Insektizid	Schopf Hygiene	pyrethrins + rapeseed oil	potato beetle, sucking insects, free-feeding butterfly caterpillars, biting insects, aphids, moth scale insects, scale insects, wolly aphids, leaf wasps, leaf-eating beetles
Raptol AF RosenSchädlingsfrei	NEUDORFF GmbH KG	pyrethrins + rapeseed oil	aphids, moth scale insects, scale insects, wolly aphids, sucking insects
Raptol SchädlingsSpray	NEUDORFF GmbH KG	pyrethrins + rapeseed oil	aphids, moth scale insects, scale insects, wolly aphids, sucking insects
Schädlingsfrei Forte	Evergreen Garden Care	pyrethrins + rapeseed oil	aphids, ground fleas, cabbage moth, lily chicken, butterfly caterpillars, cabbage white butterfly species, bedbugs, soft scale insects, scale insects, white flies
Schädlingsfrei Forte Konzentrat	Evergreen Garden Care	pyrethrins + rapeseed oil	aphids, scale insects, butterfly caterpillars
Schädlingsfrei Forte Konzentrat	Evergreen Garden Care	pyrethrins + rapeseed oil	aphids, scale insects, butterfly caterpillars, moth scale insects, white flies
Schädlingsfrei Hortex	Evergreen Garden Care	rapeseed oil	aphids, white flies, scale insects
Schädlingsfrei Naturen	Evergreen Garden Care	rapeseed oil	aphids, white flies, scale insects, sucking insects
SCHÄDLINGSFREI NATUREN AF	Evergreen Garden Care	rapeseed oil	aphids, white flies, scale insects
Schädlingsfrei Neem	Plantura GmbH	azadirachtin	potato beetle, leaf-mining insects, biting insects, sucking insects, aphids, free-feeding butterfly caterpillars, cockchafer, vine pest, white flies, fungus gnats, thrips
SCHÄDLINGSFREI ORGANIC	Schopf Hygiene	azadirachtin	potato beetle, leaf-mining insects, biting insects, sucking insects, aphids, free-feeding butterfly caterpillars, cockchafer, vine pest, white flies, fungus gnats, thrips

Schädlings-Stopp Plus	Andermatt Biogarten GmbH	azadirachtin	potato beetle, leaf-mining insects, biting insects, sucking insects, aphids, free-feeding butterfly caterpillars, cockchafer, vine pest, white flies, fungus gnats, thrips
Schildlaus-Pumpspray	Chrysal International BV	paraffin oil (CAS 8042-47-5)	scale insects, wolly aphids
Spruzit AF OrchideenSchädlingsFrei	NEUDORFF GmbH KG	pyrethrins + rapeseed oil	aphids, moth scale insects, scale insects, wolly aphids, sucking insects
Spruzit AF RosenSchädlingsFrei	NEUDORFF GmbH KG	pyrethrins + rapeseed oil	aphids, moth scale insects, scale insects, wolly aphids, sucking insects
Spruzit AF Schädlingsfrei	NEUDORFF GmbH KG	pyrethrins + rapeseed oil	aphids, moth scale insects, scale insects, wolly aphids, sucking insects
Spruzit NEEM GemüseSchädlingsfrei	NEUDORFF GmbH KG	azadirachtin	potato beetle, leaf-mining insects, biting insects, sucking insects, aphids, free-feeding butterfly caterpillars, cockchafer, vine pest, white flies, fungus gnats, thrips
Spruzit Neu	NEUDORFF GmbH KG	pyrethrins + rapeseed oil	potato beetle, sucking insects, free-feeding butterfly caterpillars, biting insects, aphids, moth scale insects, scale insects, wolly aphids, leaf wasps, leaf-eating beetles
Spruzit OrchideenSchädlingsSpray	NEUDORFF GmbH KG	pyrethrins + rapeseed oil	aphids, moth scale insects, scale insects, wolly aphids, sucking insects
Spruzit RosenSchädlingsSpray	NEUDORFF GmbH KG	pyrethrins + rapeseed oil	aphids, moth scale insects, scale insects, wolly aphids, sucking insects
Spruzit Schädlingsfrei	NEUDORFF GmbH KG	pyrethrins + rapeseed oil	potato beetle, sucking insects, free-feeding butterfly caterpillars, biting insects, aphids, moth scale insects, scale insects, wolly aphids, leaf wasps, leaf-eating beetles
Spruzit SchädlingsSpray	NEUDORFF GmbH KG	pyrethrins + rapeseed oil	aphids, moth scale insects, scale insects, wolly aphids, sucking insects
Spruzit TrauermückenFrei	NEUDORFF GmbH KG	azadirachtin	potato beetle, leaf-mining insects, biting insects, sucking insects, aphids, free-feeding butterfly caterpillars, cockchafer, vine pest, white flies, fungus gnats, thrips

SUBSTRAL SCHÄDLINGSFREI	Evergreen Garden Care	rapeseed oil	aphids, white flies, scale insects
Substral Schädlingsfrei	Evergreen Garden Care	rapeseed oil	aphids, white flies, scale insects
TrauermückenEX	Evergreen Garden Care Deutschland GmbH	azadirachtin	potato beetle, leaf-mining insects, biting insects, sucking insects, aphids, free-feeding butterfly caterpillars, cockchafer, vine pest, white flies, fungus gnats, thrips
Trauermückenfrei Neem	Plantura GmbH	azadirachtin	potato beetle, leaf-mining insects, biting insects, sucking insects, aphids, free-feeding butterfly caterpillars, cockchafer, vine pest, white flies, fungus gnats, thrips
UNIVERSAL SCHÄDLINGSFREI	Schopf Hygiene	azadirachtin	potato beetle, leaf-mining insects, biting insects, sucking insects, aphids, free-feeding butterfly caterpillars, cockchafer, vine pest, white flies, fungus gnats, thrips

Table S2. Commercially available products based on botanical insecticides in Algeria [1].

Commercial product	Active compound	Formulation	Insects targets	Company
BIOAZA 32	Azadirachtin	Emulsifiable Concentrate	Aphids; Leafminer; Thrips Mites; Ceratites; Whitefly Pine processionary	SIPCAM INAGRA (Spain)
DEFFORT	Plant Extract (4% alkaloids from Fabaceae) + 1% Mn + 1% Zn	Liquid	Pear Psyllids	ALTINCO SL (Spain)
MATRINAL	2% Oil of terpenes + Extract by *Sophora flavescens* (8% alkaloids)	Liquid	Whiteflies Tomato; Psyllids	ALTINCO SL (Spain)
SAMUILE	Végétable oil (Fatty acid)	Soluble Concentrate	Mites; Mealybugs; Aphids	AGRIPHAR S.A. (Belgium)
TUTAFORT	Limonene + Plant extract (0.1% alkaloids)	Emulsifiable Concentrate	Leafminer (*Tuta absoluta*)	ALTINCO SL (Spain)

Table S3. Plant-derived substrates and extracts with bioherbicide activity.

Plant source	Extract Type or Compound	Inhibitory Effects	Controlled weed	References
Aglaia odorata Lour.	Leaf extract	Inhibition of growth	*E. crus-galli* (L.) P.B., *L. perenne* L.	[2]
Ailanthus altissima (Mill.) Swingle	Leaf extract	Inhibition of germination and growth	*Medicago sativa* L.	[3]
Ammi visnaga (L.) Lam.	Plant extract	Inhibition of germination, growth and photosynthesi s	*Digitaria sanguinalis, Echinochloa crus-galli* (L.) P.B., *Lolium multiflorum L., Setaria italica* (L.) P. Beauv.	[4]
Artemisia vulgaris L., *Mentha spicata L.* subsp. *spicata, Ocimum basilicum* L., *Salvia officinalis* L., *Thymbra spicata* L. subsp. *spicata*	Essential oils derived from flowers and leaves	Inhibition of germination and growth	*Agrostemma githago* L., *Cardaria draba* (L.) Desv., *Chenopodium album* L., *E. crus-galli* (L.) P.B., *Reseda lutea* L.	[5]
Cymbopogon citratus (DC.) Stapf., *Micromeria fruitcosa* L., *Origanum syriacum* L.,	Essential oils	Inhibition of germination	*Amaranthus palmeri S. Watson, Brassica nigra* (L.) Koch, *Triticum asteivum* L.	[6]
Eucalyptus citriodora Hook.	Essential oil derived from leaves	Inhibition of germination	*Parthenium hysterophorus* L.	[7]
Eucalyptus spp., *Chamaecyparis lawsoniana* (A.Murray bis) Parl., *Rosmarinus officinalis* L., *Thuja occidentalis* L.	Essential oils	Act as pre-emergent and inhibit germination	*Amaranthus retroflexus* L., *Portulaca oleracea* L., *Acroptilon repens* (L.) DC.	[8]
Juglans nigra L.	Plant extract	Act as pre- and post-emergent and inhibit growth	*Conyza bonariensis* (L.) Cronquist, *C. canadensis* (L.) Cronquist.	[9]
Leptospermum scoparium J. R. Forst. & G. Forst.	Essential oil	Act as post-emergent and inhibit seed emergence	*Digitaria* spp.	[10]
Oryza sativa L.	Hull extract	Inhibition of germination	*E. crus-galli* (L.) P.B.	[11]

Table S4. Selected commercial bioherbicides.

Name	Producer	Active principle
AG optima	AVENGER Products	citrus oil (D-limonene) (55%)
Bio Unkrautfrei AF	Solabiol	pelargonic acid (33 g/L)
Bioganic Broadleaf Killer	-	clove and thyme oil (2%)
Burnout	BONIDE Products LLC	clove oil (12%)
EcoSMART®	EcoSMART Techhnologies	eugenol (5.0%)
Fast acting weed killer concentrate	AVENGER Products	citrus oil (D-limonene) (70%)
Finalsan AF UnkrautFrei Plus	NEUDORFF	pelargonic acid (86 g/L), maleinic acid hydrazide (30 g/L)
GreenMatch Ex	Marrone Bio Innovations	*Cymbopogon citratus* (50%)
Herbistop	COMPO	pelargonic acid (237.59 g/L)
Matran EC	EcoSMART Technologies	clove oil (50%)
Organic Interceptor TM	Marrone Bio Innovations	pelargonic acid (10%), pine oil (*Pinus sylvestris*)
Phydura™	Soil Technologies Corp	clove oil (8%)
RapidGo	PROGEMA GmbH	pelargonic acid (699,4 g/L)
Scythe Herbicide	Mycogen Corporation	pelargonic acid (57%), C6-C12 fatty acids (3%)
Weed Zap®	JH Biotech Inc	clove oil (45%), cinnamon oil (45%)

Table S5. Commercially available plant-derived substrates and extracts with fungicide activity.

**Commercial name**	**Plant source**	**Extract Type or Compound**	**Controlled disease**	**References**
Bla-S™	-	-	Rice blast fungus	[12]
Carvone™	Caraway and dill seeds	Volatile compounds	Pathogens affecting stored crops	[13]
Delvolan™	-	-	Fungal diseases of ornamental plants	[14]
E-Rase™	*Simmondsia california*	Jojoba oil	#	[14]
Fungastop	*Mentha spicata*	Mint oil	Powdery mildews, *Botrytis cinerea*	Technical Center of Organic Agriculture-Tunisia: http://www.ctab.nat.tn
Fungastop™ and Armorex™	-	-	Powdery mildews	[14]
GC-3™	*Gossypium hirsutum* + *Allium sativum*	Cottonseed oil + Garlic extract	#	[14]
Kasugamin™	-	-	Rice blast fungus	[15]
Mildiomycin™	-	-	Powdery mildews	[15]
Milsana ®	*Reynoutria sachalinensis* (giant knotweed)	Ethanol extract	Powdery mildews	[16]
Promax™	*Thymus vulgaris*	thyme oil	#	[15]
Sporan™	*Rosmarinus officinalis*	Rosemary oil	#	[14]
Timorex Gold ®	*Melaleuca alternifolia*	Aqueous extract	Powdery and downy mildews, rust, early and late blight diseases	[14]
Trilogy™	*Azadirachta indica*	Neem oil	#	[14]
Validacin™	-	-	Rhizoctonia root rot	[15]

#, for organic farming application.

References

1. DPVCT. Index Des Produits Phytosanitaires À Usage Agricole 2015; Direction de la protection des végétaux et des contrôles techniques: 2015; p. 216.
2. Kato-Noguchi, H.; Suzuki, M.; Noguchi, K.; Ohno, O.; Suenaga, K.; Laosinwattana, C. A potent phytotoxic substance in *Aglaia odorata* Lour. *Chem. Biodivers.* **2016**, *13*, 549–554.
3. Tsao, R.; Romanchuk, F.E.; Peterson, C.J.; Coats, J.R. Plant growth regulatory effect and insecticidal activity of the extracts of the Tree of Heaven (*Ailanthus altissima* L.). *BMC Ecol.* **2002**, *2*, 1–6.
4. Travaini, M.L.; Sosa, G.M.; Ceccarelli, E.A.; Walter, H.; Cantrell, C.L.; Carrillo, N.J.; Dayan, F.E.; Meepagala, K.M.; Duke, S.O. Khellin and visnagin, furanochromones from *Ammi visnaga* (L.) Lam., as potential bioherbicides. *J. Agric. Food Chem.* **2016**, *64*, 9475–9487.
5. Onen, H.; Ozer, Z.; Telci, I. Bioherbicidal effects of some plant essential oils on different weed species. *Z. Pflanzenkrankh. Pflanzenschutz, Sonderh.* **2002**, *18*, 597–606.
6. Dudai, N.; Poljakoff-Mayber, A.; Mayer, A.; Putievsky, E.; Lerner, H. Essential oils as allelochemicals and their potential use as bioherbicides. *J. Chem. Ecol.* **1999**, *25*, 1079–1089.
7. Singh, H.; Batish, D.; Setia, N.; Kohli, R. Herbicidal activity of volatile oils from *Eucalyptus citriodora* against *Parthenium hysterophorus*. *Ann. Appl. Biol.* **2005**, *146*, 89–94.
8. Ramezani, S.; Saharkhiz, M.J.; Ramezani, F.; Fotokian, M.H. Use of essential oils as bioherbicides. *J. Essent. Oil Bear. Plants* **2008**, *11*, 319–327.
9. Shrestha, A. Potential of a black walnut (*Juglans nigra*) extract product (NatureCur®) as a pre-and post-emergence bioherbicide. *J. Sustain. Agric.* **2009**, *33*, 810–822.
10. Dayan, F.E.; Howell, J.L.; Marais, J.P.; Ferreira, D.; Koivunen, M. Manuka oil, a natural herbicide with preemergence activity. *Weed Sci.* **2011**, *59*, 464–469.
11. Ahn, J.K.; Chung, I.M. Allelopathic potential of rice hulls on germination and seedling growth of barnyardgrass. *Agron. J.* **2000**, *92*, 1162–1167.
12. Zaker, M.; Mosallanejad, H. Antifungal activity of some plant extracts on *Alternaria alternata*, the causal agent of Alternaria leaf spot of potato. *Pak. J. Biol. Sci.* **2010**, *13*, 1023–1029.
13. Moezelaar, R.; Braam, C.; Zomer, J.; Gorris, L.G.M.; Smid, E.J. Volatile plant metabolites for postharvest crop protection. In *Modern fungicides and antifungal compounds II*; Lyr,

H., Russell, P.E., Dehne, H.W., Sisler, H.D., Eds.; Andover: Intercept Limited, 1999; pp. 453–467.

14. Zaker, M. Natural plant products as eco-friendly fungicides for plant diseases control-A review. *Agriculturists* **2016**, *14*, 134–141.
15. Dayan, F.E.; Cantrell, C.L.; Duke, S.O. Natural products in crop protection. *Bioorg. Med. Chem.* **2009**, *17*, 4022–4034.
16. Daayf, F. The effects of plant extracts of *Reynoutria sachalinensis* on powdery mildew development and leaf physiology of long English cucumber. *Plant Dis.* **1995**, *79*, 577.
